# Brg1 restrains the pro-inflammatory properties of ILC3s and modulates intestinal immunity

**DOI:** 10.1038/s41385-020-0317-3

**Published:** 2020-07-01

**Authors:** Xinyi Qi, Jinxin Qiu, Jiali Chang, Yan Ji, Qi Yang, Guoliang Cui, Liming Sun, Qian Chai, Jun Qin, Ju Qiu

**Affiliations:** 1 CAS Key Laboratory of Tissue Microenvironment and Tumor, Shanghai Institutes for Biological Sciences (SIBS), Chinese Academy of Sciences (CAS), Shanghai Jiao Tong University School of Medicine (SJTUSM), 200031 Shanghai, China; 2 CAS Key Laboratory of Tissue Microenvironment and Tumor, Shanghai Institute of Nutrition and Health, Shanghai Institutes for Biological Sciences, Chinese Academy of Sciences, University of Chinese Academy of Sciences, 200031 Shanghai, China; 3 Department of Immunology and Microbial Disease, Albany Medical College, Albany, NY 12208 USA; 4 T Cell Metabolism Group (D140), German Cancer Research Center (DKFZ), Im Neuenheimer Feld 280, 69120 Heidelberg, Germany; 5 Faculty of Biosciences, Heidelberg University, 69120 Heidelberg, Germany; 6 State Key Laboratory of Cell Biology, CAS Center for Excellence in Molecular Cell Science, Shanghai Institute of Biochemistry and Cell Biology, Chinese Academy of Sciences, University of Chinese Academy of Sciences, 200031 Shanghai, China; 7 Key Laboratory of Infection and Immunity, Institute of Biophysics, Chinese Academy of Sciences, 100101 Beijing, China

## Abstract

Group 3 innate lymphoid cells (ILC3s), a subset of the innate lymphoid cells, are abundantly present in the intestine and are crucial regulators of intestinal inflammation. Brg1 (Brahma-related gene 1), a catalytic subunit of the mammalian SWI-SNF-like chromatin-remodeling BAF complex, regulates the development and function of various immune cells. Here, by genetic deletion of Brg1 in ILC3s (*Smarca4*^*ΔILC3*^), we prove that Brg1 supports the differentiation of NKp46^+^ILC3s by promoting the T-bet expression in NKp46^*−*^ILC3s, which facilitates the conversion of NKp46^*−*^ILC3s to NKp46^+^ILC3s. Strikingly, *Smarca4*^*ΔILC3*^ mice of the *Rag1*^*−/−*^ background develop spontaneous colitis accompanied with increased GM-CSF production in ILC3s. By construction of a mixed bone marrow chimeric system, we demonstrate that Brg1 enhances T-bet and inhibits GM-CSF expression in ILC3s through a cell-intrinsic manner. Blockade of GM-CSF ameliorates colitis in *Rag1*^*−/−*^*Smarca4*^*ΔILC3*^ mice, suggesting that the suppression of GM-CSF production from ILC3s by Brg1 serves as a critical mechanism for Brg1 to restrain intestinal inflammation. We have further demonstrated that Brg1 binds to the *Tbx21* and *Csf2* gene locus in ILC3s, and favors the active and repressive histones modifications on gene locus of *Tbx21* and *Csf2* respectively. Our work reveals the essential role of Brg1 in intestinal immunity by regulating ILC3s.

## Introduction

Group 3 innate lymphoid cells (ILC3s) belong to the ILC lineages, which are composed of subsets of ILC3s that lack T- and B-cell antigen specific receptors.^1–3^ ILCs are abundantly present in mucosal tissues and play important roles in the initiation, progression, and resolution of inflammation. ILCs mirror T helper (Th) cells in the expression of transcription factors and functional cytokines.^1^ ILC3s are similar to Th17 cells, which are featured by the expression of retinoic acid-related orphan receptor gamma t (RORγt) and production of IL-17 and IL-22.^3^

In both humans and mice, ILC3s are found to be localized in the intestinal lamina propria and function as “double-edge sword” in intestinal inflammatory diseases.^4^ On one hand, ILC3s are important for immune defense against intestinal bacterial and viral infections.^5–7^ On the other hand, ILC3s have been shown to be pathogenic in innate colitis.^8,9^ The dual effect of ILC3s in intestinal inflammation is partially mediated through differential functions of cytokines produced by ILC3s. Except for IL-17 and IL-22, ILC3s have been reported to be able to produce IFN-γ, TNF-α, and GM-CSF.^10–12^ IL-22 is critical for inhibiting the expansion of pathogens by promoting antimicrobial peptides production from intestinal epithelial cells (IECs).^13^ Both IL-17 and IL-22 are important for maintenance of intestinal epithelial integrity by facilitating the regeneration of IECs.^14,15^ However, overt production of IL-17 and IL-22 could result in accumulation of neutrophils, which facilitates pathogen clearance but exacerbates tissue damage.^9,16^ Besides IL-17 and IL-22, IFN-γ produced by ILC3s has been suggested to contribute to the pathogenesis of innate colitis.^8,9,17^

GM-CSF has been shown to be detrimental in several human autoimmune diseases and in mouse studies, such as experimental autoimmune encephalomyelitis, rheumatoid arthritis, and inflammatory bowel diseases (IBD).^9,11,18–20^ ILC3s have been shown to be a major source for GM-CSF in the intestine.^12,21^ Microbiota-triggered cytokines, including IL-1β, tumor necrosis factor-like cytokine 1A (TL1A) and IL-23 produced by intestinal mononuclear phagocytes (MNPs), enhances GM-CSF production from ILC3s.^11,12,22^ Under the steady state, ILC3-derived GM-CSF is essential for the generation of CD4^+^ regulatory T cells (Tregs) by maintaining tissue-resident MNPs.^12^ Nevertheless, pathological level of GM-CSF produced by ILC3s causes mobilization of ILC3s within the intestinal lamina propria and exacerbates inflammation.^9,11^ GM-CSF production by colitogenic T cells is required for the recruitment of eosinophils to the intestine and exacerbates colitis.^18^ Therefore, an optimal dose of GM-CSF production from ILC3s is important for the balancing intestinal immune responses, and elaborating the molecular regulation of GM-CSF expression by intestinal ILC3s is critical.

According to the expression of surface molecules and functional diversity, ILC3s are classified into three subsets, which are NKp46^+^CCR6^*−*^ILC3s, NKp46^*−*^CCR6^+^ILC3s, and NKp46^*−*^CCR6^−^ILC3s.^10^ NKp46^+^CCR6^*−*^ILC3s (also characterized as NKp46^+^ILC3s) express T-bet and IFN-γ, whereas the NKp46^−^CCR6^+^ILC3s are T-bet^−^ and produce few IFN-γ.^10^ Studies on cell ontogeny of mouse ILC3s suggest that NKp46^+^ILC3s appear in the intestine at about 2 weeks of age and could be originated from a subset of T-bet^+^NKp46^*−*^CCR6^−^ ILC3s, a process dependent on Notch signaling and the transcription factor aryl hydrocarbon receptor (Ahr).^10,23–26^ NKp46^+^ILC3s may lose the expression of RORγt and become ILC1s, another subset of ILCs characterized by T-bet expression and IFN-γ production.^1,27,28^ Such processes could be driven by pro-inflammatory cytokines, including IL-12, IL-15, and IL-18, and is considered to contribute to the progress of IBD.^3,29^ Therefore, it is important to unveil the molecular mechanisms underlying the distribution, maintenance, and conversion of ILC3 subsets.

Brg1 (encoded by *Smarca4*) is a catalytic subunit of the mammalian BAF complex, which has ATPase activity and regulates the structure of chromatin by nucleosome positioning.^30,31^ BAF has been found to cooperate with transcription factors and bind to the chromatin, which usually promotes, but sometimes suppresses the transcription of target genes.^32–34^ BAF has been shown to facilitate the formation of accessible regions by eviction of polycomb repressive complexes from the chromatin and result in loss of H3K27me3, which causes the repressive state of the chromatin.^35,36^ Consistently, mutation of *Smarca4* has been reported to be associated with loss of H3K27ac, which marks active promoters or enhancers and gene transcription.^37^ However, in cases in which gene transcription is suppressed by BAF, Brg1 could be associated with and required for the formation of H3K27me3.^34^ Previous studies have demonstrated that Brg1 is important for the development, differentiation, and function of various cell types, including immune cells, such as B cells and helper T cells.^38–41^ We thus hypothesize that the development and function of ILC3s could be regulated by Brg1, which would have significant impact on intestinal inflammatory diseases.

By crossing *Smarca4*^*flox/flox*^ mouse to *Rorc-cre* mouse, we ablate Brg1 expression in ILC3s. We have also bred the *Smarca4*^*flox/flox*^*Rorc-cre* mouse to *Rag1*^*−/−*^ mouse to determine the adaptive immunity-independent function of Brg1 on ILC3s. We have found that Brg1 is required for the development of NKp46^+^ILC3s by supporting T-bet expression in NKp46^*−*^ILC3s and by promoting the conversion of NKp46^*−*^ILC3s to NKp46^+^ILC3s driven by Notch signaling. In mouse of the *Rag1*^*−/−*^ background, Brg1 limits the homeostatic expansion of ILC3s and suppresses the pathogenicity of ILC3s to cause colitis by inhibiting GM-CSF production. We have further demonstrated that Brg1 enhances the expression of *Tbx21*, whereas suppresses the expression of *Csf2* by differentially modulating the H3K27me3 and H3K27ac modifications on the locus of target genes. Our work has revealed a critical role of Brg1 in intestinal inflammation by regulating ILC3s.

## Results

### Brg1 constrains the homeostasis of intestinal ILC3s but sustains NKp46^+^ILC3s

**Fig. 1:**
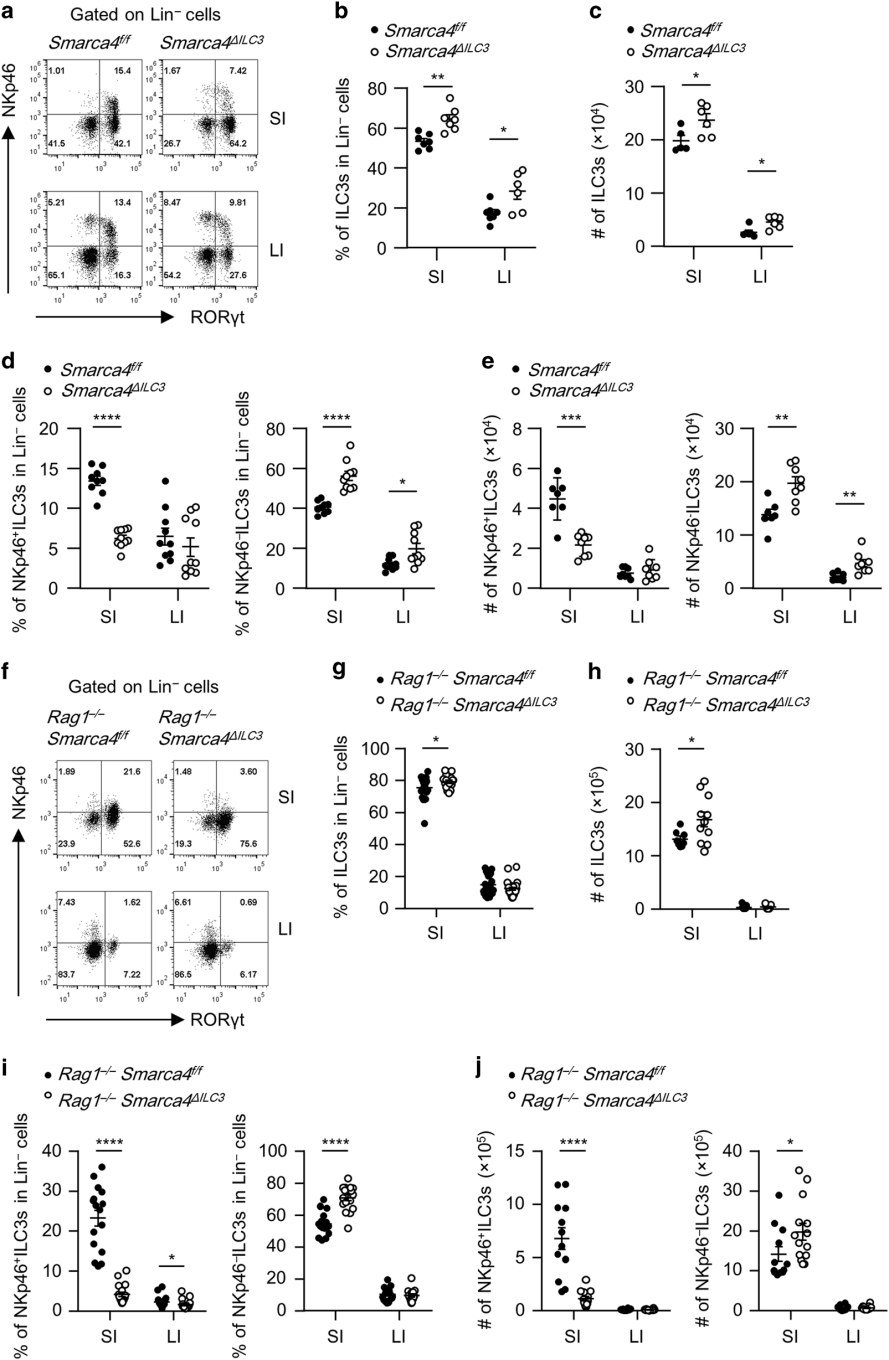
Brg1 constrains the homeostasis of intestinal ILC3s but sustains NKp46^+^ILC3s. **a**–**e** Small (SI) and large (LI) intestinal lamina propria lymphocytes (LPLs) were isolated from *Smarca4*^*f/f*^ or *Smarca4*^*f/f*^*Rorc-cre* (*Smarca4*^*ΔILC3*^) mice. (*n* = 5–10; representative of four experiments). **a** Representative flow cytometry plots for NKp46^+^ILC3s (Lin^*−*^RORγt^+^NKp46^+^) and NKp46^*−*^ILC3s (Lin^−^RORγt^+^NKp46^−^) gated on Lin^−^ cells. **b** Frequencies of ILC3s (Lin^−^RORγt^+^) in Lin^−^ cells. **c** Total numbers of ILC3s. **d** Frequencies of NKp46^+^ILC3s and NKp46^−^ILC3s in Lin^*−*^ cells. **e** Total numbers of NKp46^+^ILC3s and NKp46^−^ILC3s. **f**–**j** SI and LI LPLs were isolated from *Rag1*^*−/−*^*Smarca4*^*f/f*^ or *Rag1*^−*/−*^*Smarca4*^*f/f*^*Rorc-cre* (*Rag1*^*−/*−^*Smarca4*^*ΔILC3*^) mice. (*n* = 16–23; representative of 12 experiments). **f** Representative flow cytometry plots for NKp46^+^ILC3s and NKp46^−^ILC3s gated on Lin^*−*^ cells. **g** Frequencies of ILC3s in Lin^−^ cells. **h** Total numbers of ILC3s. **i** Frequencies of NKp46^+^ILC3s and NKp46^*−*^ILC3s in Lin^−^ cells. **j** Total numbers of NKp46^+^ILC3s and NKp46^*−*^ILC3s. **a**–**j** Data are represented as means ± SEM. Error bars show SEM. *P < 0.05; **P < 0.01; ***P < 0.001; ****P < 0.0001. Lin^−^, CD3ε^*−*^B220^*−*^CD11b^*−*^CD11c^−^.

We firstly analyzed the mRNA expression of *Smarca4*, which encodes Brg1, in purified subsets of ILC3s and lineage (Lin)^−^non-ILC3s by real-time RT-PCR (Fig. S1a). We detected comparable level of *Smarca4* mRNA expression in NKp46^+^ILC3s, NKp46^*−*^ILC3s and Lin^−^nonILC3s from both the small intestine and large intestine (Fig. S1a). To investigate the function of Brg1 in ILC3s, we crossed *Smarca4*^*flox/flox*^ mouse to *Rorc-cre* mouse to specifically delete Brg1 in ILC3s and T cells.^42,43^ We found the percentages of ILC3s among Lin^*−*^ cells and absolute numbers of ILC3s were increased in *Smarca4*^*flox/flox*^*Rorc-cre* (*Smarca4*^*ΔILC3*^) mice compared with littermate controls *Smarca4*^*flox/flox*^ (*Smarca4*^*f/f*^) mice in both small and large intestinal lamina propria lymphocytes (LPLs) (Fig. 1a–c). As a note, we used *Smarca4*^*f/f*^ mice as controls for *Smarca4*^*f/f*^*Rorc-cre* including mice crossed to *Rag1*^*−/*−^ and *Rorc*^*gfp/+*^ background. When analyzing the functional subsets of ILC3s, we observed a reduction in the proportion of NKp46^+^ILC3s among Lin^*−*^ cells and absolute numbers of NKp46^+^ILC3s in the small intestinal LPLs of *Smarca4*^*ΔILC3*^ mice, whereas the percentages and numbers of NKp46^−^ILC3s were increased in both small and large intestinal LPLs of *Smarca4*^*ΔILC3*^ mice compared with control group (Fig. 1a, d, e). The above data indicate that Brg1 supports the maintenance of small intestinal NKp46^+^ILC3s and may suppress the expansion of NKp46^−^ILC3s.

A previous study has shown that deletion of Brg1 in T cells results in sporadic systemic autoimmunity in aged mice, mainly due to impaired function of Tregs.^39^ In *Smarca4*^*ΔILC3*^ mice lacking Brg1 in both ILC3s and T cells, we detected elevated levels of activated CD4^+^T cells as well as increased CD103 expression in Tregs, which was consistent with what has been found in *Smarca4*^*flox/flox*^*CD4-cre* or *Smarca4*^*flox/flox*^*Foxp*^*YFP-cre*^ mice (Fig. S1b–e).^39^ This potential pro-inflammatory environment in *Smarca4*^*ΔILC3*^ mice may affect intestinal ILC3s. We then firstly analyzed ILC3s in *Smarca4*^*flox/flox*^*CD4-cre* mice which had Brg1 deficiency in only T cells.^42^ We observed slight reduction in the percentages of total ILC3s and NKp46^−^ILC3s in large intestinal LPLs of *Smarca4*^*flox/flox*^*CD4-cre* mice, which was inconsistent with changes of ILC3s in *Smarca4*^*ΔILC3*^ mice (Fig. S1f–h). These data suggest that phenotypes of ILC3s in *Smarca4*^*ΔILC3*^ mice are not caused by indirect effect of Brg1 deficiency in T cells. To further eliminate the impact of Brg1-deficient T cells on ILC3s, we crossed *Smarca4*^*flox/flox*^*Rorc-cre* mouse to *Rag1*^*−/−*^ mouse which lacked T and B cells. Efficient deletion of Brg1 in ILC3s of *Rag1*^*–/–*^*Smarca4*^*flox/flox*^*Rorc-cre* (*Rag1*^*−/−*^*Smarca4*^*ΔILC3*^) mice was confirmed by real-time RT-PCR and flow cytometry (Fig. S1i, j). In *Rag1*^*−/−*^*Smarca4*^*ΔILC3*^ mice, we consistently observed enhanced percentages and absolute numbers of ILC3s in the small intestine compared with littermate controls (Fig. 1f–h). Similar to *Smarca4*^*ΔILC3*^ mice of the immunocompetent background, percentages of NKp46^+^ILC3s was decreased and percentages of NKp46^−^ILC3s were increased among Lin^−^ cells from small intestinal LPLs of *Rag1*^*−/*−^*Smarca4*^*ΔILC3*^ mice (Fig. 1f, i). The above trend was also reflected by changes of absolute numbers of NKp46^+^ILC3s and NKp46^*−*^ILC3s in the small intestine (Fig. 1j). In the large intestine, percentages of NKp46^+^ILC3s in Lin^−^ cells were reduced in *Rag1*^*−/−*^*Smarca4*^*ΔILC3*^ mice (Fig. 1f, i). NKp46^*−*^ILC3s could be further categorized to CCR6^+^ILC3s and NKp46^*–*^CCR6^*–*^ILC3s. We found that the percentages and numbers of NKp46^*–*^CCR6^*–*^ILC3s but not CCR6^+^ILC3s were increased in the small intestine of *Rag1*^*−/−*^*Smarca4*^*ΔILC3*^ mice compared with controls (Fig. S1k–m). Percentages of CCR6^+^ILC3s but not absolute numbers were reduced in the large intestine of *Rag1*^*−/−*^*Smarca4*^*ΔILC3*^ mice (Fig. S1k, m). Therefore, NKp46^−^CCR6^−^ILC3s but not CCR6^+^ILC3s account for the upregulation of small intestinal NKp46^−^ILC3s in *Rag1*^*−/*−^*Smarca4*^*ΔILC3*^ mice. Together, our data suggest that Brg1 is required for the maintenance of intestinal NKp46^+^ILC3s. Moreover, Brg1 constrains the homeostasis of intestinal ILC3s, which is especially obvious in the small intestine independently of the adaptive immune system.

### Ablation of Brg1 in ILC3s unleashes the proliferative capacity of ILC3s in a cell-intrinsic manner

**Fig. 2:**
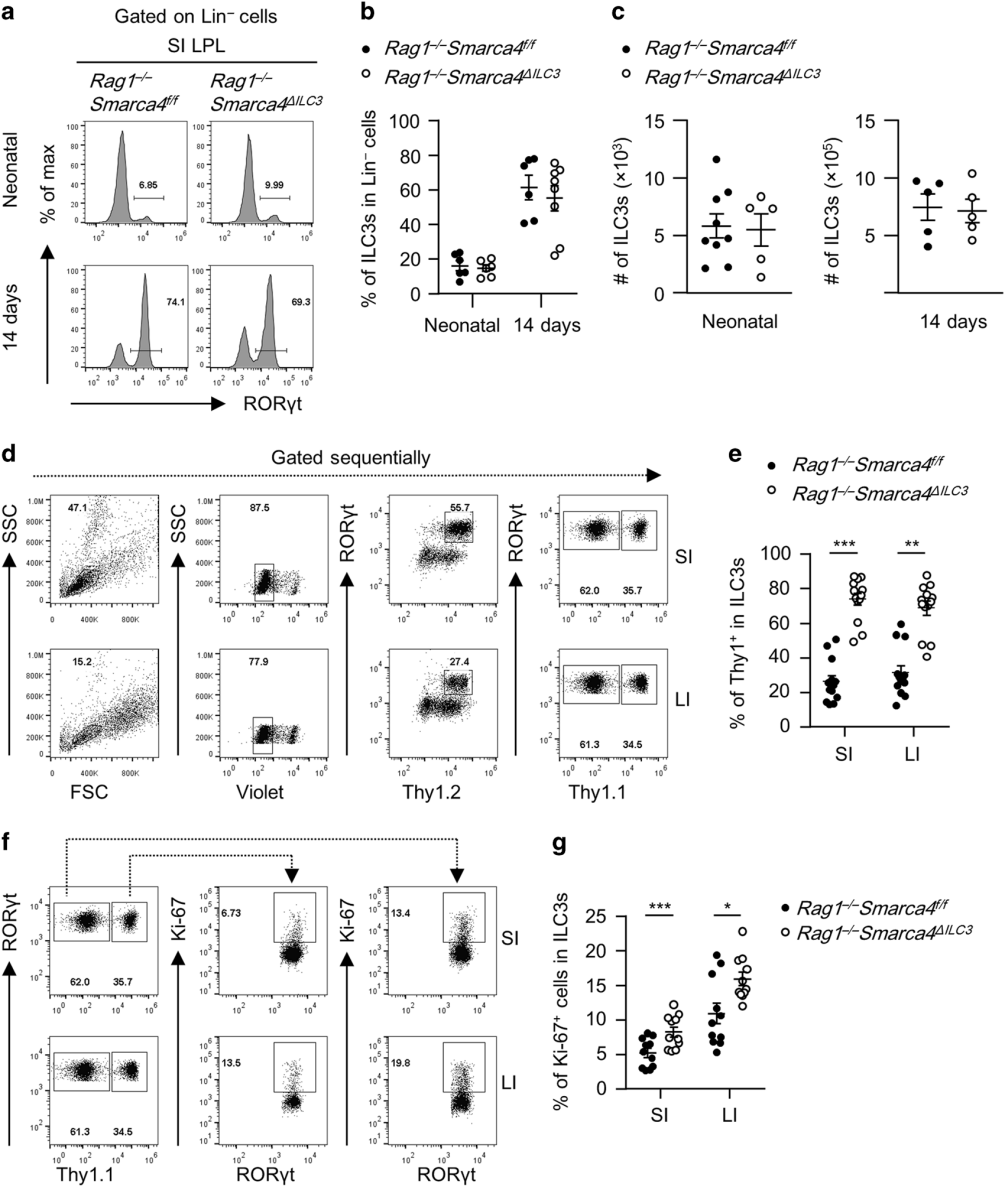
Ablation of Brg1 in ILC3s unleashes the proliferative capacity of ILC3s in a cell-intrinsic manner. **a**–**c** SI LPLs were isolated from neonatal or 14-day-old *Rag1*^*−/*−^*Smarca4*^*f/f*^ or *Rag1*^−*/−*^*Smarca4*^*ΔILC3*^ mice. (*n* = 5–9; Representative of five experiments). **a** Histogram plots of flow cytometry analysis for RORγt frequency gated on Lin^−^ cells. **b** Frequencies of ILC3s in Lin^−^ cells. **c** Total numbers of ILC3s. **d**–**g** Mixed bone marrow chimeric mice were constructed as in Fig. S2 and sacrificed for analyses 6 weeks later. (*n* = 11–13; representative of six experiments) (**d**) FACS gating strategies of ILC3s in SI and LI LPLs of mixed bone marrow chimeric mice. **e** Frequencies of ILC3s from indicated hosts among all ILC3s identified by the congenic marker Thy1. **f** Representative flow cytometry plots showing gating strategy for analyzing Ki-67 expression in ILC3s from indicated hosts identified by the congenic marker Thy1 based on total Thy1^+^ILC3s gate. **g** Frequencies of Ki-67^+^ cells in ILC3s. **a**–**g** Data are represented as means ± SEM. Error bars show SEM. *P < 0.05; **P < 0.01; ***P < 0.001. Lin^*−*^, CD3ε^*−*^B220^−^CD11b^−^CD11c^−^.

To investigate the kinetics of Brg1 in restricting the homeostasis of intestinal ILC3s, we analyzed intestinal ILC3s from *Rag1*^*−/−*^*Smarca4*^*ΔILC3*^mice and littermate controls during ontogeny. We found comparable levels of percentages and absolute numbers of ILC3s in Lin^−^ cells in the small intestine of newborn and 2-week-old *Rag1*^*−/−*^*Smarca4*^*ΔILC3*^ mice, suggesting the increase of ILC3s is attributed to an accumulative effect during growth rather than an incidence from birth (Fig. 2a–c). RORγt has been reported to be expressed by a few non-ILC3 cell types.^44,45^ To stringently evaluate the function of Brg1 on ILC3s per se, we generated mixed bone marrow chimeric mice by mixing equal numbers of bone marrow cells from *Rag1*^*−/*−^*Smarca4*^*f/f*^ and *Rag1*^*−/*−^*Smarca4*^*ΔILC3*^mice with separate congenic markers, and transferring them to half-lethally irradiated *Rag2*^*−/−*^*Il2rg*^*−/−*^ mice which were deficient for adaptive immune cells and ILCs (Fig. S2).^46,47^ This also built a competitive environment for the growth of ILC3s from the donors. In the above system, we found Brg1-deficient ILC3s were significantly more abundant than controls among total ILC3s in both the small and large intestine (Fig. 2d, e). Consistently, we observed enhanced rate of proliferation in ILC3s from *Rag1*^*−/*−^*Smarca4*^*ΔILC3*^ donors compared to controls, as was indicated by increased percentages of Ki-67^+^ cells (Fig. 2f, g).^48^ Together, the data suggest that Brg1 constrains the homeostasis of ILC3s by suppression of cell proliferation through a cell-intrinsic mechanism.

### Brg1 facilitates the conversion of NKp46^−^ILC3s to NKp46^+^ILC3s by promoting T-bet expression

**Fig. 3:**
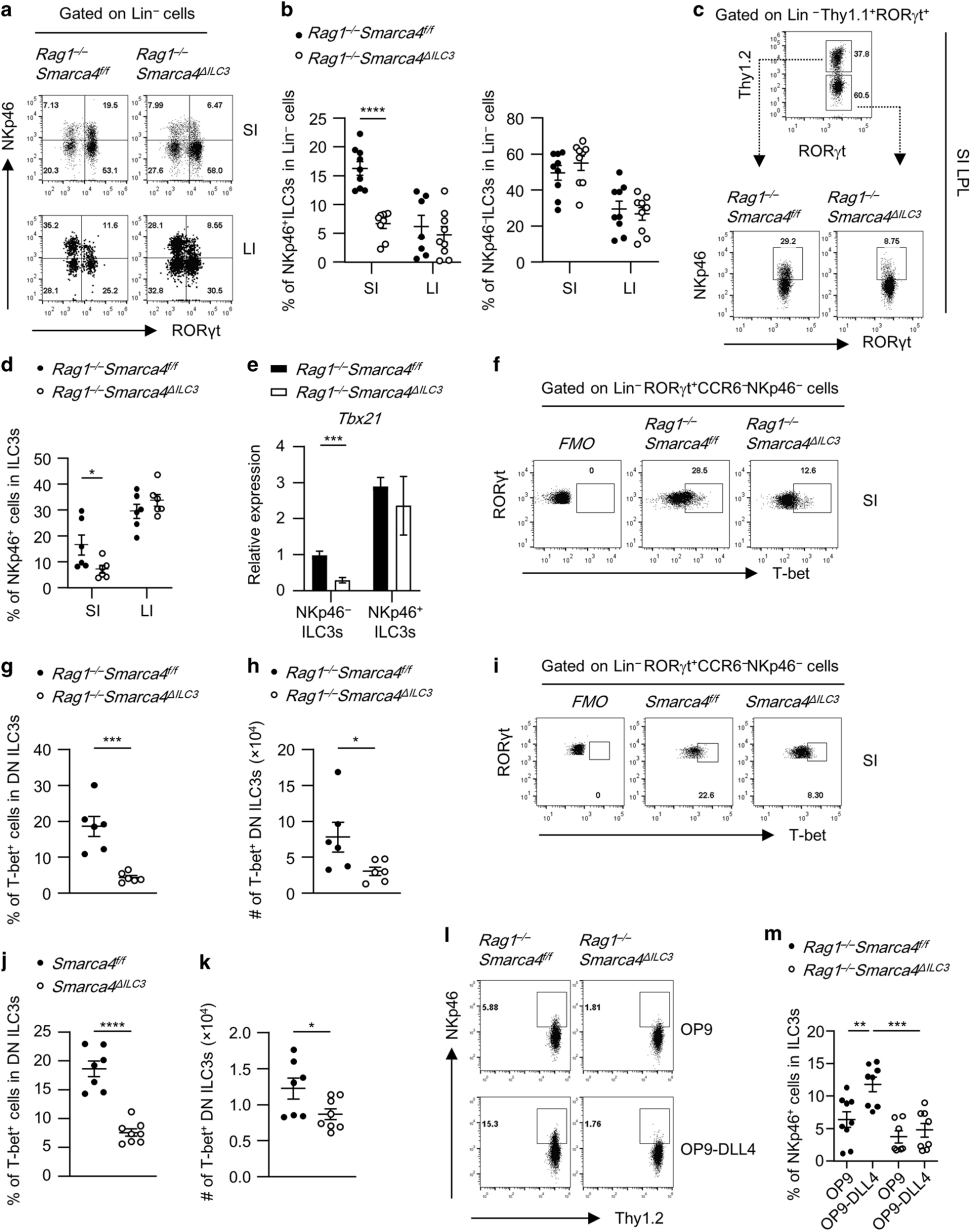
Brg1 facilitates the conversion of NKp46^−^ILC3s to NKp46^+^ILC3s by promoting T-bet expression. **a**, **b** SI and LI LPLs were isolated from 14-day-old *Rag1*^*−/−*^*Smarca4*^*f/f*^ or *Rag1*^*−/−*^*Smarca4*^*ΔILC3*^ mice. (*n* = 9; representative of three experiments). **a** Representative flow cytometry plots for NKp46^+^ILC3s (Lin^−^RORγt^+^NKp46^+^) and NKp46^*−*^ILC3s (Lin^−^RORγt^+^NKp46^*−*^) gated on Lin^*−*^ cells. **b** Frequencies of NKp46^+^ILC3s and NKp46^*−*^ILC3s in Lin^*–*^ cells. **c**, **d** Mixed bone marrow chimeric mice were constructed as in Fig. S2, except for that three of the biological repeats were constructed using *Rag1*^*−/−*^ instead of *Rag2*^−*/−*^*Il2rg*^−*/*−^ mice as recipients. Mice were sacrificed for analyses 6 weeks later. (*n* = 6; representative of two experiments). **c** Representative flow cytometry plots showing percentages of NKp46^+^ILC3s among total Thy1^+^ILC3s in SI LPL from indicated donors. **d** Frequencies of NKp46^+^ILC3s in ILC3s from the small intestine of *Rag1*^*−/*−^*Smarca4*^*f/f*^ or *Rag1*^−*/−*^*Smarca4*^*ΔILC3*^ donor mice. **e** Relative expression of *Tbx21* in NKp46^+^ILC3s (CD45^low^Thy1.2^high^NKp46^+^) and NKp46^−^ILC3s (CD45^low^Thy1.2^high^NKp46^−^) sorted from the SI LPLs of *Rag1*^−*/*−^*Smarca4*^*f/f*^ or *Rag1*^*−/−*^*Smarca4*^*ΔILC3*^ mice (*n* = 6, representative of two independent experiments). **f**–**h** SI LPLs were isolated from *Rag1*^−*/*−^*Smarca4*^*f/f*^ or *Rag1*^−*/−*^*Smarca4*^*ΔILC3*^ mice. **f** Representative flow cytometry plots for T-bet^+^ILC3s gated on Lin^−^RORγt^+^NKp46^−^CCR6^−^ ILC3s. **g** Frequencies of T-bet^+^ILC3s in Lin^*−*^RORγt^+^NKp46^−^CCR6^−^ ILC3s. **h** Total numbers of NKp46^−^CCR6^−^T-bet^+^ ILC3s. **i**–**k** SI LPLs were isolated from *Smarca4*^*f/f*^ or *Smarca4*^*ΔILC3*^ mice. (*n* = 6; representative of two experiments). **i** Representative flow cytometry plots for T-bet^+^ILC3s gated on Lin^*−*^RORγt^+^NKp46^−^CCR6^−^ ILC3s. **j** Frequencies of T-bet^+^ILC3s in Lin^−^RORγt^+^NKp46^*−*^CCR6^*−*^ ILC3s. **k** Total numbers of NKp46^*−*^CCR6^−^T-bet^+^ ILC3s. **l**, **m** Sorted NKp46^−^ILC3s were cultured on OP9-GFP or OP9-delta like canonical Notch ligand 4 (DLL4) for 7 days. (*n* = 7–9; representative of three experiments). **l** Representative flow cytometry plots for NKp46^+^ILC3s (NKp46^+^CD45^low^Thy1.2^high^) on ILC3s (CD45^low^Thy1.2^high^ cells). **m** Frequencies of NKp46^+^ILC3s in ILC3s. **a**–**m** Data are represented as means ± SEM. Error bars show SEM. *P < 0.05; **P < 0.01; ***P < 0.001; ****P < 0.0001. Lin^−^, CD3ε^−^B220^*−*^CD11b^−^CD11c^−^. DN ILC3s, Lin^−^RORγt^+^NKp46^−^CCR6^−^ ILC3s.

The reduction of NKp46^+^ILC3s was readily to be detected in the small intestine of *Rag1*^−*/*−^*Smarca4*^*ΔILC3*^ mice at the age of 2 weeks, when NKp46^+^ILC3s starts to appear (Fig. 3a, b).^10^ The reduction of NKp46^+^ILC3s among total ILC3s in the absence of Brg1 compared with controls was also observed in the small intestine using the system of mixed bone marrow chimeric mice, in which Brg1-deficient and sufficient ILC3s existed in the same environment, further suggesting that Brg1 supports the maintenance of NKp46^+^ILC3s in a cell-intrinsic manner (Figs. 3c, d and S2).

By breeding *Rag1*^*−/−*^*Smarca4*^*ΔILC3*^ mice to *Rorc*^*gfp/gfp*^ mice, we generated the *Rag1*^*−/−*^*Smarca4*^*ΔILC3*^*Rorc*^*gfp/+*^ mice with GFP indicating the RORγt expression.^49^ Reduced percentages of NKp46^+^ILC3s were consistently observed in *Rag1*^*−/−*^*Smarca4*^*ΔILC3*^*Rorc*^*gfp/+*^ mice compared to *Rag1*^*−/−*^*Smarca4*^*f/f*^*Rorc*^*gfp/+*^ mice (Fig. S3a, b). Taking the advantage of GFP representing RORγt expression, we analyzed the cell apoptosis ex vivo in subsets of ILC3s. We found no difference in the rate of cell apoptosis in NKp46^+^ILC3s from both the small and large intestine of *Rag1*^*−/−*^*Smarca4*^*ΔILC3*^*Rorc*^*gfp/+*^ mice compared to controls. Intriguingly, we found increased percentages of apoptotic cells in the large intestine of *Rag1*^*−/−*^*Smarca4*^*ΔILC3*^*Rorc*^*gfp/+*^ mice (Fig. S3a, c). This was probably due to a pro-inflammatory environment in the large intestine of *Rag1*^*−/−*^*Smarca4*^*ΔILC3*^*Rorc*^*gfp/+*^ mice (described in the next section). Previous research has demonstrated that NKp46^+^ILC3s may lose the expression of RORγt and become ILC1s.^27,28^ To test the impact of Brg1 on the conversion of ILC3s to ILC1s in the absence of Brg1, we crossed *Smarca4*^*ΔILC3*^ mouse to *Rosa26*^*LSL–YFP/+*^ mouse to track the fate of ILC3s.^50^ About 80% of ILC3s were YFP^+^ cells and this ratio was similar in mice with or without specific Brg1 deficiency in ILC3s (Fig. S3d). We found percentages of ILC1s among Lin^*−*^YFP^+^ cells (consisting both current and fate converted ILC3s) were comparable between *Rosa26*^*LSL−YFP/+*^*Smarca4*^*f/f*^*Rorc-cre* and *Rosa26*^*LSL−YFP/+*^*Smarca4*^*f/+*^*Rorc-cre* mice in both the small and large intestine, suggesting similar conversion rate of ILC3s to ILC1s in the presence or absence of Brg1 (Fig. S3e, f). The above data suggest the reduction of NKp46^+^ILC3s in *Rag1*^*−/−*^*Smarca4*^*ΔILC3*^ mice is less likely to be due to different level of cell survival or the conversion of ILC3s to ILC1s.

Previous studies have shown that NKp46^+^ILC3s can be differentiated from T-bet^+^NKp46^*–*^ILC3s, a process that requires Notch signaling.^10,23–26^ Indeed, mRNA expression of *Tbx21*, which encodes T-bet, was significantly lower in NKp46^*−*^ILC3s of *Rag1*^−*/*−^*Smarca4*^*ΔILC3*^ mice compared with controls, whereas mRNA expression of *Tbx21* in NKp46^+^ILC3s was comparable (Fig. 3e). Specifically, NKp46^−^CCR6^−^ILC3s rather than NKp46^−^CCR6^+^ILC3s have been reported to possess the potential to generate NKp46^+^ILC3s.^10^ Consistently, reduced proportion of T-bet^+^ cells were found among NKp46^−^CCR6^*−*^ILC3s in *Rag1*^*−/*−^*Smarca4*^*ΔILC3*^ mice than controls (Fig. 3f, g), and absolute numbers of T-bet^+^NKp46^−^CCR6^−^ILC3s were decreased in the small intestine of *Rag1*^*−/−*^*Smarca4*^*ΔILC3*^ mice (Fig. 3h). The reduction of T-bet^+^ cells among NKp46^−^CCR6^−^ILC3s was similarly found in the small intestine of immunocompetent *Smarca4*^*ΔILC3*^ mice compared with controls (Fig. 3i–k). In addition, NKp46^*–*^ILC3s purified from the small intestine of *Rag1*^*−/−*^*Smarca4*^*ΔILC3*^ mice had a defect of converting into NKp46^+^ILC3s driven by Notch ligand compared with control group in an in vitro culture system (Fig. 3l, m). Together, these data suggest that Brg1 is required for the differentiation of NKp46^+^ILC3s by facilitating the conversion of NKp46^*−*^ILC3s to NKp46^+^ILC3s.

### *Rag1*^*−/−*^*Smarca4*^*ΔILC3*^ mice develop spontaneous colitis due to the pathogenicity of ILCs

**Fig. 4:**
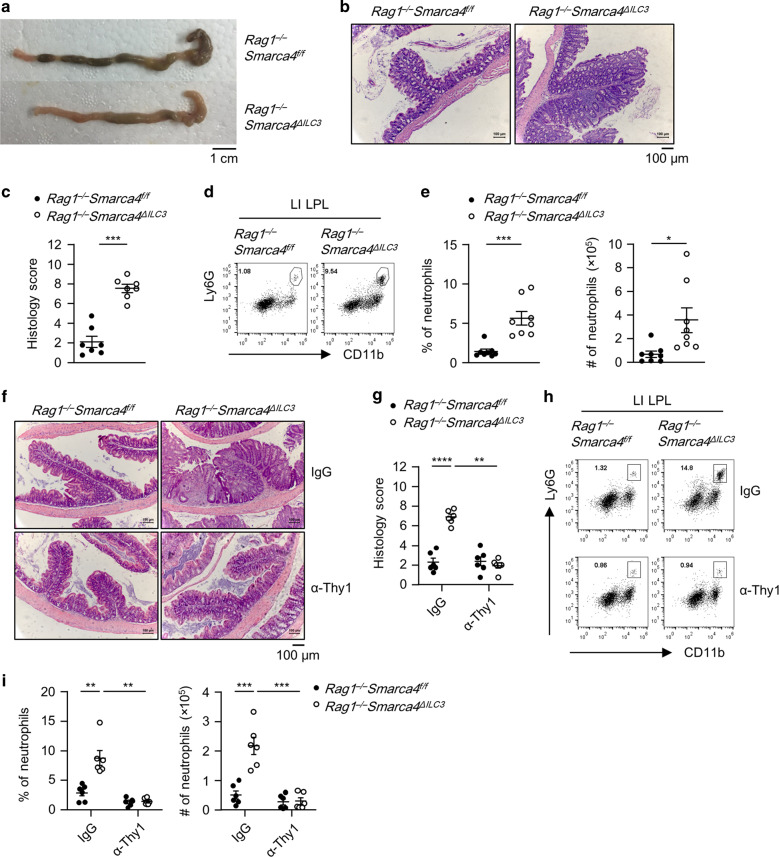
*Rag1*^*−/*−^*Smarca4*Δ^*ILC3*^ mice develop spontaneous colitis due to the pathogenicity of ILCs. **a**–**e** 6–8-week-old *Rag1*^*−/−*^*Smarca4*^*f/f*^ or *Rag1*^*−/−*^*Smarca4*^*ΔILC3*^ mice were sacrificed for analyses. (*n* = 7–8; representative of three experiments). **a** Representative image of colon from mice with indicated genotypes. Scale bar is 1 cm. **b** Representative H&E staining of colon sections (magnification ×10) and histological score. Scale bar is 100 μm. **c** Histological scores of colon sections. **d** Representative flow cytometry plots for neutrophils (CD11b^+^Ly6G^+^) in LI LPLs. **e** Frequencies and total numbers of lamina propria infiltrating neutrophils. **f**–**i** 4-week-old *Rag1*^*−/−*^*Smarca4*^*f/f*^ or *Rag1*^*−/−*^*Smarca4*^*ΔILC3*^ were treated with IgG or α-Thy1.2 antibody for 14 days and sacrificed for analyses. (*n* = 6; Representative of two experiments). **f** Representative image of H&E staining of colon sections (magnification ×10). Scale bar is 100 μm. **g** Histological scores of colon sections. **h** Representative flow cytometry plots for neutrophils (CD11b^+^Ly6G^+^) in LI LPLs. **i** Frequencies and total numbers of neutrophils in LI LPLs. **a**–**i** Data are represented as means ± SEM. Error bars show SEM. *P < 0.05; **P < 0.01; ***P < 0.001; ****P < 0.0001. Lin^−^, CD3ε^−^B220^−^CD11b^−^CD11c^−^.

*Rag1*^−*/−*^*Smarca4*^*ΔILC3*^ mice developed spontaneous colitis at 6–8 weeks of age, characterized by opportunistic diarrhea, rectal prolapse, and rectal bleeding (Fig. S4a). Histological analyses showed that *Rag1*^*−/*−^*Smarca4*^*ΔILC3*^ mice had loosened stool (Fig. 4a). Hematoxylin and eosin (H&E) staining of colon sections revealed pathology of chronic colitis in *Rag1*^*−/−*^*Smarca4*^*ΔILC3*^ mice featured by epithelial hyperplasia and increased infiltration of leukocytes (Fig. 4b, c). As a note, no inflammation was observed in the small intestine of *Rag1*^–*/–*^*Smarca4*^*ΔILC3*^ mice, as was indicted by the increased percentages and absolute numbers of neutrophils in the large but not small intestinal LPLs of *Rag1*^*−/−*^*Smarca4*^*ΔILC3*^mice compared to controls, implying microbiota may be involved in the onset of colitis (Figs. 4d, e, S4b, c). The percentages and absolute numbers of eosinophils were similar in the large intestine of *Rag1*^*−/−*^*Smarca4*^*ΔILC3*^ mice and controls (Fig. S4d, e). Since the deficiency of Brg1 was restricted to ILC3s in *Rag1*^−*/−*^*Smarca4*^*ΔILC3*^ mice, we reasoned that the colitis was caused by the pathogenicity of Brg1-deficient ILC3s. To test this hypothesis, we depleted ILC3s from *Rag1*^*−/−*^*Smarca4*^*ΔILC3*^ mice using α-Thy1 neutralizing antibody.^51^ Strikingly, colitis was significantly ameliorated upon treatment of α-Thy1 antibody, as indicated by improved histological scores, decreased percentages, and absolute numbers of neutrophils in the large intestine of α-Thy1-treated *Rag1*^*−/−*^*Smarca4*^*ΔILC3*^ mice compared with IgG-treated group (Fig. 4f–i). Together, the above data suggest that Brg1-deficient ILC3s are pathogenic and can cause spontaneous colitis in the absence of the adaptive immune system. Notably, we observed no signs of colitis in *Smarca4*^*ΔILC3*^ mice by the age of 8 weeks as indicated by similar level of neutrophil infiltration, in spite of increased T-cell activation probably due to defective function of Tregs in the absence of Brg1 (Figs. S1b, c, S4f, g). In addition, we found that the percentages of Tc17 cells (CD3^+^CD8^+^RORγt^+^ cells) in CD8+ T cells, percentages of Th17 cells (CD3^+^CD8^+^RORγt^+^ cells) in CD4+ T cells, as well as absolute numbers of Tc17 and Th17 cells were comparable between *Smarca4*^*ΔILC3*^ mice and *Smarca4*^*f/f*^ mice (data not shown). We reasoned that the inflammation triggered by Brg1-deficient ILC3s may be suppressed by the adaptive immune system, which has been shown to prevent overt innate responses.^52^

### Effect of Brg1 on the transcriptome profile of ILC3s

**Fig. 5:**
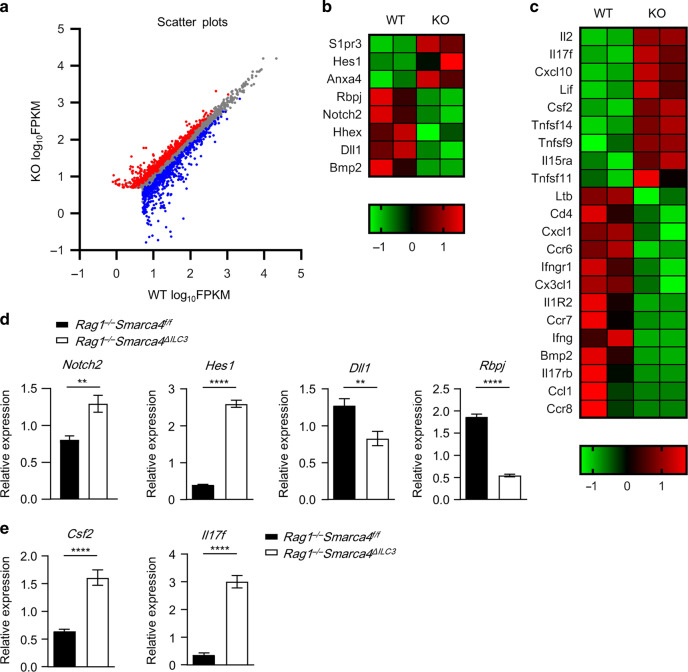
Effect of Brg1 on the transcriptome profile of ILC3s. **a**–**c** Mixed bone marrow chimeric mice were constructed as in Fig. S2 using Thy1.2/1.2*Rag1*^*−/−*^*Smarca4*^*f/f*^ and Thy1.1/1.2*Rag1*^*−/−*^*Smarca4*^*ΔILC3*^ donors. Biological duplicates of small intestinal ILC3s from the Brg1-deficient and sufficient donor origins were sorted for RNA-seq analysis. **a** Scattered plots showing Log_10_FPKM value of genes with FPKM > 5. Genes significantly upregulated (red) or downregulated (blue) for more than 2^0.5^-fold in ILC3s from *Rag1*^−*/*−^*Smarca4*^*ΔILC3*^ donors compared with controls were marked. **b** Heatmap of significantly changed genes (FPKM > 15) belong to the Notch signaling pathway (GO0007219). **c** Heatmap of significantly changed genes belonging to the categories of cytokines, cytokine receptor, chemokine, and chemokine receptors (KEGG_mmu04060). **d**, **e** ILC3s were sorted from the small intestine of *Rag1*^*−/−*^*Smarca4*^*f/f*^ or *Rag1*^−*/−*^*Smarca4*^*ΔILC3*^ mice. (*n* = 6; representative of two experiments). **d** Expression of *Dll1*, *Rbpj*, and *Notch2* was analyzed by real-time RT-PCR. **e** Expression of *Il17f* and *Csf2* was analyzed by real-time RT-PCR. Data are represented as means ± SEM. Error bars show SEM. **P < 0.01; ****P < 0.0001.

To search for cell-intrinsic targets of Brg1 in ILC3s in a genome-wide scope, we isolated Brg1-deficient ILC3s and control ILC3s from the small intestine of mixed bone marrow chimeric mice as described above and performed transcriptome sequencing (Fig. S2). Within the expression of genes significantly changed above 2^0.5^-fold, downregulated genes (575) outnumbered upregulated genes (493) in Brg1-deficient ILC3s compared with controls, consistent with the previously reported role of Brg1 in favoring activation of gene transcription (Supplementary Data 1 and Fig. 5a). Gene ontology analysis revealed that downregulated genes in Brg1-deficient ILC3s were enriched in immune responses, immune system process, inflammatory response, and regulation of cell proliferation pathways (Fig. S5a, b). And genes with increased expression in Brg1-deficient ILC3s were overrepresented in pathways of regulation of ion transmembrane transport, lipid metabolism process, and response to virus (Fig. S5a, c). Our data have suggested that Brg1 promotes the conversion of NKp46^*−*^ILC3s to NKp46^+^ILC3s driven by Notch signaling, and Brg1 suppresses the pathogenicity of ILC3s to cause colitis. We therefore further screened the significantly changed genes with average FPKM value higher than 15 in either group and belonged to the categories of Notch signaling pathways and cytokine/cytokine receptors/chemokines/chemokines receptors (Fig. 5b, c). Real-time RT-PCR was performed to confirm on the expression of the key candidates in ILC3s purified from *Rag1*^*−/*−^*Smarca4*^*ΔILC3*^ mice and control mice. Expression of *Notch2* and *Hes1* was increased, whereas and *Dll1* and *Rbpj* was decreased in Brg1-deficient ILC3s compared with controls (Fig. 5d). The discrepancy trend of change in *Notch2* expression analyzed by RNA-seq compared with real-time RT-PCR was likely to be due to differential sources of ILC3s purified from mixed bone marrow chimeric mice or directly from adult *Rag1*^*−/−*^*Smarca4*^*ΔILC3*^ versus *Rag1*^*−/−*^*Smarca4*^*f/f*^ mice separately. Among the significantly changed Notch signaling-related genes, cell-intrinsic expression of *Rbpj* as the classic Notch co-activator, has been clearly demonstrated to be crucial for the development of NKp46^+^ILC3s.^23^ The decreased expression of *Rbpj* in Brg1-deficient ILC3s may contribute to the defective differentiation of NKp46^+^ILC3s. Strikingly, we confirmed the upregulated expression of *Il17f* and *Csf2*, two cytokines reported to be pathogenic in colitis in ILC3s from *Rag1*^−*/*−^*Smarca4*^*ΔILC3*^mice (Fig. 5e).^11,18,53^

### Deletion of Brg1 endows ILC3s with pro-inflammatory property of producing GM-CSF

**Fig. 6:**
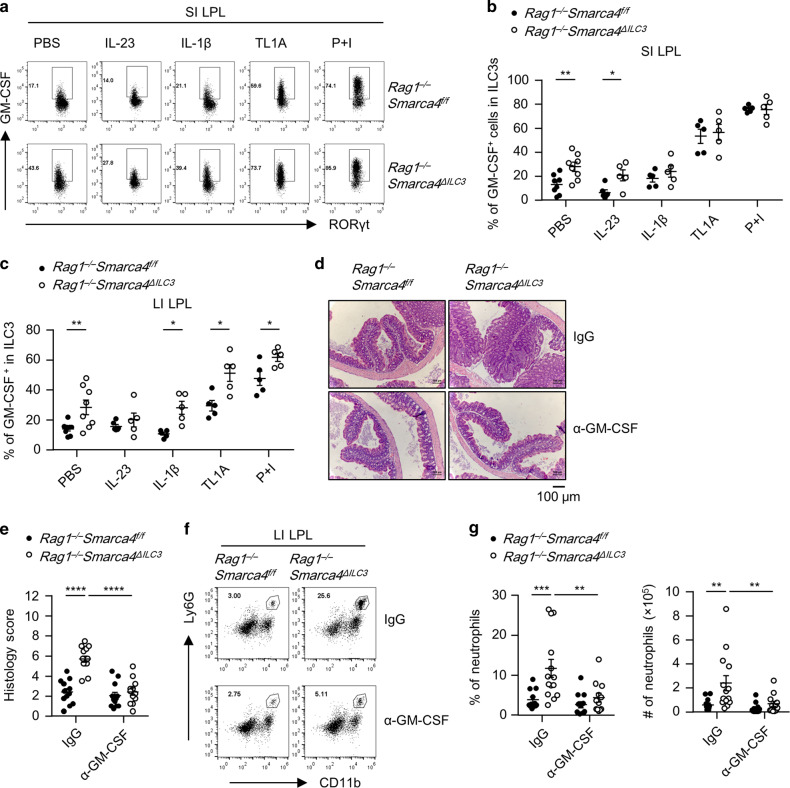
Deletion of Brg1 endows ILC3s with pro-inflammatory property of producing GM-CSF. **a**–**c** LPLs isolated from *Rag1*^−*/−*^*Smarca4*^*f/f*^ or *Rag1*^*−/*−^*Smarca4*^*ΔILC3*^ mice were stimulated with PBS, IL-23 (10 ng/ml), IL-1β (10 ng/ml) or TL1A (10 ng/ml) overnight or PMA plus ionomycin for 3 h prior to analyses. (*n* = 5–8; representative of four experiments). **a** Representative flow cytometry plots for GM-CSF (**a**) expression gated on ILC3s (CD45^low^Thy1.2^high^RORγt^+^) in SI LPLs. **b**, **c** Frequencies of GM-CSF^+^ cells in ILC3s from SI (**b**) and LI (**c**) LPLs. **d**–**g** 4–6-week-old *Rag1*^*−/−*^*Smarca4*^*f/f*^ or *Rag1*^*−/−*^*Smarca4*^*ΔILC3*^ mice were treated with 250 μg of IgG or α-GM-CSF per mouse every 3 days for 14 days before analyses. (*n* = 12–14; representative of four experiments). **d** Representative H&E staining of colon sections (magnification ×10). Scale bar is 100 μm. **e** Histological scores of colon sections. **f** Representative flow cytometry plots for neutrophils (CD11b^+^Ly6G^+^) gated on live cells in LI LPLs. **g** Frequencies and total numbers of neutrophils in LI LPLs. **a**–**g** Data are represented as means ± SEM. Error bars show SEM. *P < 0.05; **P < 0.01; ***P < 0.001. Lin^*−*^, CD3ε^−^B220^*−*^CD11b^−^CD11c^−^.

We then treated the small and large intestinal LPLs with or without a series of pro-inflammatory cytokines, which are usually present in the microenvironment of colitis, and analyzed the expression of GM-CSF and IL-17F at the protein level in Brg1-deficient or sufficient ILC3s.^12,18,22^ We found the expression of GM-CSF in ILC3s was significantly higher in Brg1-deficient ILC3s compared with controls in both the small and large intestine under neutral conditions (Fig. 6a–c). This trend was consistently maintained when small intestinal LPLs were stimulated with IL-23, and when large intestinal LPLs were stimulated with IL-1β, TL1A, and phorbol 12-myristate 13-acetate (PMA) plus ionomycin (Fig. 6b, c). Moreover, in immunocompetent *Smarca4*^*ΔILC3*^mice, GM-CSF expression from ILC3s was also found to be higher in the small and large intestine under neutral condition, as well as in the large intestine when LPLs were treated with IL-1β (Fig. S6a–c). In the mixed bone marrow chimeric system (Fig. S2), where ILC3s from *Rag1*^*−/−*^*Smarca4*^*ΔILC3*^ mice and controls were resided in the same environment, we consistently observed higher level of GM-CSF expression from Brg1-deficient ILC3s than from control ILC3s in the presence and absence of inflammatory stimulus except for small intestinal LPLs stimulated with PMA plus ionomycin (Fig. S6d–f). The above data collectively suggest that Brg1 suppresses the production of GM-CSF by ILC3s through a cell-intrinsic mechanism.

ILC3s have been shown to be a dominant source of GM-CSF in the intestine.^11,12^ Therefore, the enhanced GM-CSF production from Brg1-deficient ILC3s was likely to be a key colitogenic factor for colitis in *Rag1*^*−/−*^*Smarca4*^*ΔILC3*^ mice. To test this hypothesis, we treated mice with α-GM-CSF neutralizing antibodies in vivo. Strikingly, we found blockade of GM-CSF significantly ameliorated the colitis of *Rag1*^*−/−*^*Smarca4*^*ΔILC3*^ mice, as was indicated by histological analyses showing reduced epithelial hyperplasia, leukocyte infiltration, and histology scores (Fig. 6d, e). Consistently, percentages and absolute numbers of neutrophils were decreased upon treatment with α-GM-CSF in the large intestine of *Rag1*^−*/−*^*Smarca4*^*ΔILC3*^mice compared with IgG group, whereas infiltration of eosinophils were similar (Figs. 6f, g, S6g, h). Therefore, we conclude Brg1 suppresses intestinal inflammation by restraining the GM-CSF production from ILC3s.

Expression of IL-17F was comparatively low in the small intestinal ILC3s from both *Rag1*^*−/−*^*Smarca4*^*ΔILC3*^ mice and controls under the neutral condition (Fig. S7a–c). However, when small intestinal LPLs were treated with pro-inflammatory stimulus, and when the large intestinal LPLs were treated with or without inflammatory stimulus, IL-17F expression from Brg1-deficient ILC3s was observed to be higher than from control ILC3s (Fig. S7a–c). Nevertheless, treatment of IL-17F neutralizing antibody did not show ameliorative effect on colitis of *Rag1*^*−/−*^*Smarca4*^*ΔILC3*^ mice, as was indicated by similar intestinal pathology revealed by H&E staining, similar histology scores, and comparable infiltration of neutrophils in *Rag1*^*−/−*^*Smarca4*^*ΔILC3*^ mice treated with or without α-IL-17F (Fig. S7d–g). Therefore, the colitogenic pathogenicity of Brg1-deficient ILC3s is predominantly mediated by enhanced production of GM-CSF but not IL-17F.

### Brg1 regulates the epigenetic status of *Csf2* and *Tbx21* in ILC3s

**Fig. 7:**
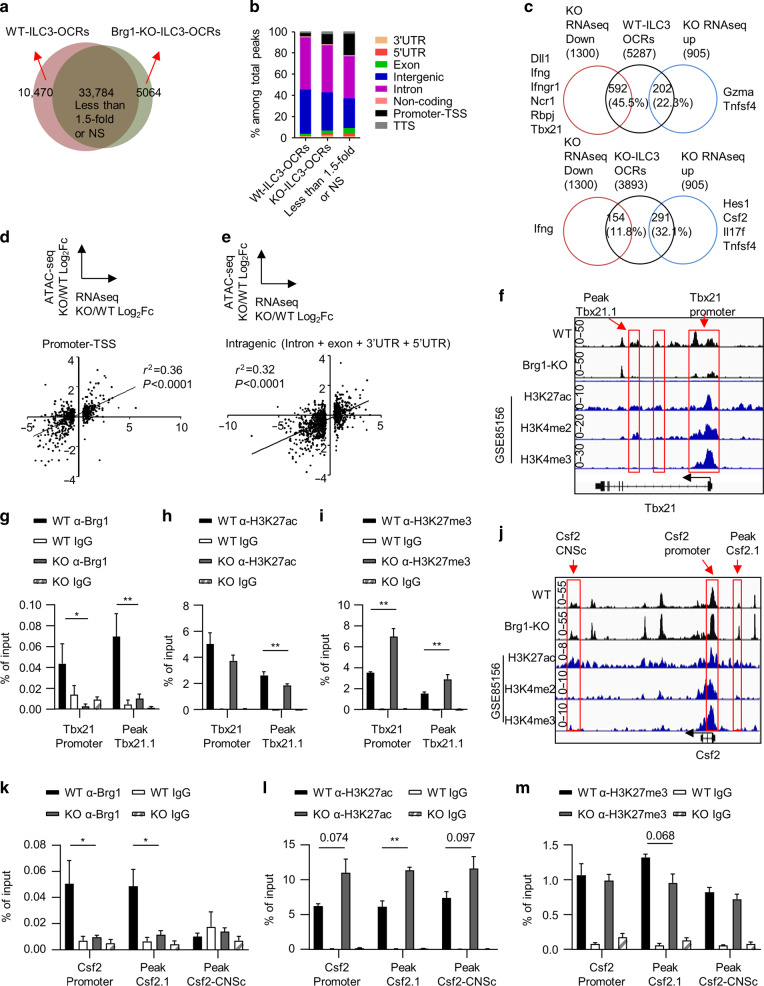
Brg1 regulates the epigenetic status of *Csf2* and *Tbx21* in ILC3s. **a**–**e**, **f**, **j** ILC3s (Thy1.2^high^CD45^low^ cells) were purified from the small intestine of *Rag1*^*−/−*^*Smarca4*^*f/f*^ or *Rag1*^*−/*−^*Smarca4*^*ΔILC3*^ mice and ATAC-seq was performed and analyzed. **a** Venn gram depicting numbers of WT-ILC3-OCRs, Brg1-KO-ILC3-OCRs with differential expression higher than 1.5-fold and the rest identified peaks. Definition of WT or Brg1-KO-ILC3-OCRs was described in methods. NS means no significant difference. **b** Proportions of different peak types among total peaks (see “Methods” for definition) were shown. **c** OCR correlated genes were overlapped with genes, the expression of which was upregulated or downregulated in Brg1-deficient ILC3s for higher than 1.2-fold from RNA-seq analysis performed in Fig. 5. Percentages of overlapped genes in total number of genes with upregulated or downregulated expression were calculated. Representative overlapped genes were listed. **d**, **e** Correlation analysis was performed on Log2 fold of gene expression (Brg1-deficient over wild-type ILC3s) and the average Log2 fold in accessibility of OCRs distributed at promoters (**d**) or intragenic regions (**e**). *R*^2^ indicate correlation coefficient. **f**, **j** Integrated visualization of ATAC-seq peaks at the Tbx21 (**f**) or Csf2 (**j**) locus in WT or Brg1-deficient ILC3s, together with published ChIP-seq data of small intestinal ILC3s (GSE85156). Red boxes highlight representative OCRs. **g**–**i**, **k**–**m** ILC3s from SI LPLs of *Rag1*^−*/−*^*Smarca4*^*f/f*^ and the *Rag1*^*−/−*^*Smarca4*^*ΔILC3*^ mice were sorted for ChIP-qPCR analysis (*n* = 3–8; representative of four experiments). The amount of input DNA and pull-down DNA was quantified by real-time RT-PCR. Enriched associations of Brg1, H3K27ac, and H3K27me3 at the *Tbx21* locus (**g–i**) and *Csf2* locus (**k**–**m**) relevant to input was calculated and shown. (**g**–**I**, **k**–**m**) Data are represented as means ± SEM. Error bars show SEM. *P < 0.05; **P < 0.01.

As one of the central component of the BAF complex, Brg1 plays an essential role in chromatin remodeling.^30,31^ We then performed assay for transposase-accessible chromatin with high throughput sequencing (ATAC-seq) with ILC3s sorted from the small intestine of *Rag1*^*−/*−^*Smarca4*^*f/f*^ or *Rag1*^*−/−*^*Smarca4*^ΔILC3^ mice. We identified statistically differential open chromatin regions (OCRs) in wild-type and Brg1-deficient ILC3s respectively according to DEseq2 analysis with a cutoff value of 1.5-fold (Fig. 7a). 10,470 peaks were more accessible in wild-type ILC3s and were identified as “WT-ILC3-OCRs”, whereas only 5064 “Brg1-KO-ILC3-OCRs” were found (Fig. 7a and Supplementary Data 2). Consistently, significantly more motifs were identified from WT-ILC3-OCRs, including Tbx21 binding motif (Supplementary Data 3). This suggests the chromatin status was generally less accessible in ILC3s in the absence of Brg1. Majority of WT or Brg1-KO-ILC3-OCRs were distributed in intron and intergenic regions (Fig. 7b). An integrated analysis of ATAC-seq and RNA-seq (with a cutoff value of 1.2-fold) revealed that genes with significantly reduced expression in Brg1-deficient ILC3s had more overlap with WT-ILC3-OCRs (592) rather than Brg1-KO-ILC3-OCRs (154) (Fig. 7c). And slightly more Brg1-KO-ILC3-OCRs (291) than WT-ILC3-OCRs (202) overlapped with genes of increased expression in Brg1-deficient ILC3s (Fig. 7c). Among them, chromatins of fundamental genes regulating the development of NKp46^+^ILC3s, including *Rbpj*, *Dll1*, and *Tbx21*, were less accessible without Brg1 (Fig. 7c). And locus of pro-inflammatory genes, including *Csf2* and *Il17f*, were more accessible in Brg1-deficient ILC3s (Fig. 7c). Therefore, Brg1 promotes the differentiation of NKp46^+^ILC3s and restrains the pro-inflammatory properties of ILC3s by positively and negatively regulate the chromatin accessibility of target genes respectively.

We noticed that the accessibility along the same gene locus could be regulated bidirectionally by Brg1, such as *Ifng* and *Tnfsf4* (Fig. 7c). We then analyzed the correlation of the fold change in gene expression and an average fold in the accessibility of differential OCRs belonging to different regions. Compared with accessibility at the intergenic regions of gene locus, accessibility of the promoter and intragenic regions had higher level of positive correlation with the relative fold of mRNA expression, as was indicated by the correlation coefficient (Figs. 7d, e and S8a). Brg1 differentially regulates target genes in a promotive or inhibitory manner, which could be reflected with histone features as H3K27ac and H3K27me3 at the promoters or enhancers.^54^ Distribution of distal enhancers varies dramatically among different cell types.^55^ Visualization of representative target genes, including *Ifng*, *Ifngr1*, *Ncr1*, *Il17f*, *Tbx21*, and *Csf2*, with aligned comparison to previous published data on intestinal ILC3s showed that differential OCRs were overlapped with or adjacent to histone modifications indicative of promoters (H3K4me3), active transcription (H3K27ac), or potential regulatory regions (H3K4me2) such as enhancers (Figs. 7f, j, S8b–e).^56^ Using chromatin immunoprecipitation coupled with quantitative PCR (ChIP Q-PCR) analysis, we found strong associations of Brg1 with the promoter and region close to peak Tbx21.1, which were also WT-ILC3-OCRs (Fig. 7f, g). Moreover, there was an increase of H3K27me3 at the above two regions and a decrease in H3K27ac near peak Tbx21.1 without Brg1 (Fig. 7h, i), suggesting that Brg1 drives the transcription of Tbx21 by opening up and activating the *Tbx21* gene locus.

Although chromatin accessibility was enhanced at Csf2 without Brg1, we detected significant association of Brg1 with *Csf2* promoter and regions close to peak Csf2.1, which was more accessible in Brg1-deficient ILC3s (Fig. 7j, k). Meanwhile, enhanced H3K27ac was found at the above two regions, suggesting them to be active promoter and enhancers. Interesting, activity of Csf2-CNSc, which has been reported to regulate transcription of *Csf2* in T cells,^57^ was increased as indicated by elevated H3K27ac, though no association of Brg1 and Csf2-CNSc was detected (Fig. 7l). Meanwhile, H3K27me3 level adjacent to peak Csf2.1 was reduced without Brg1 (Fig. 7m). Our data suggest that Brg1 binds to *Csf2* locus directly and inhibits the transcription activity of *Csf2* by downregulating active histone modifications. Loss of Brg1 increases the accessibility of *Csf2* locus likely due to recruitment of transcriptional repressors, which is one of the previously proposed regulatory manner of the SWI/SNF complex.^58^

## Discussion

In this study, we have investigated the role of Brg1 on the development and function of ILC3s. We have found that Brg1 is required for the development of NKp46^+^ILC3s by promoting the conversion of NKp46^−^ILC3s to NKp46^+^ILC3s. In addition, Brg1 inhibits the homeostatic proliferation of ILC3s and the pathogenicity of ILC3s to cause colitis by suppressing GM-CSF production.

We found that *Rag1*^*−/−*^*Smarca4*^*ΔILC3*^ mice developed spontaneous colitis at 6–8 weeks of age, which was caused by enhanced production of GM-CSF from Brg1-deficient ILC3s. In immunocompetent *Smarca4*^*ΔILC3*^ mice lacking Brg1 in both ILC3s and T cells, no discernable symptoms of colitis occurred before 8 weeks, although proportions of activated T cells was increased probably due to impaired function of Tregs.^39^ It is likely that the pathogenicity of ILC3s was inhibited by the adaptive immune system in immunocompetent *Smarca4*^*ΔILC3*^ mice.^52^ Firstly, numbers of ILCs were less in immunocompetent mice compared to *Rag1*^*−/*−^ mice.^59^ Therefore, even though GM-CSF production from *Smarca4*^*ΔILC3*^ mice was higher than controls, it may not mount a pathogenic level to cause colitis. Secondly, pro-inflammatory properties of myeloid cells, which are critical in exacerbation of colitis, could be inhibited by the adaptive immune cells.^52^ For instance, α-CD40 induces colitis only in immunocompromised mice.^60,61^ Another example is that spontaneous colitis happened in *Rag1*^*−/−*^*Tbx21*^−*/−*^ mice (TRUC mice) but not in *Tbx21*^*−/*−^ mice.^8,62^ However, it is likely that deficiency of Brg1 in ILC3s and Tregs collectively contribute to possible intestinal inflammation in aged *Smarca4*^*ΔILC3*^ mice. Since the role of Brg1 on ILC3s and T cells could not be dissected in *Smarca4*^*ΔILC3*^ mice, we did not analyze immune responses in aged *Smarca4*^*ΔILC3*^ mice. In the future, it would be interesting to investigate the role of Brg1 on ILC3s in immunocompetent environment using *Rag1*^*−/−*^*Smarca4*^*ΔILC3*^ mice reconstituted with adaptive immune cells.

Colitis in *Rag1*^*−/−*^*Smarca4*^*ΔILC3*^ mice was featured by infiltration of neutrophils but not eosinophils. Intriguingly, a previous study has suggested that GM-CSF is required for the infiltration of eosinophils but not neutrophils during colitis.^18^ Consistently, we observed a reduction of eosinophils after blockade of GM-CSF in *Rag1*^*−/−*^*Smarca4*^*ΔILC3*^ mice, as well as a trend towards a reduction of eosinophils in *Rag1*^*−/−*^*Smarca4*^*f/f*^ mice. It is likely that enhanced level of GM-CSF works collectively with other cytokines derived from ILC3s or non-ILC3s to regulate the infiltration of neutrophils and eosinophils in *Rag1*^*−/*−^*Smarca4*^*ΔILC3*^ mice. A striking decrease of neutrophils accompanied amelioration of colitis was observed in *Rag1*^−*/−*^*Smarca4*^*ΔILC3*^ but not control mice after blockade of GM-CSF, suggesting that GM-CSF is the prerequisite, though may not be a unique factor in causing the disease.

We have shown that Brg1 suppresses the homeostatic expansion of ILC3s in the intestine. This was reflected more obviously in the small intestine but not in the large intestine of *Rag1*^*−/−*^*Smarca4*^*ΔILC3*^ mice under the steady state. In fact, a trend toward a decrease in the percentage of ILC3s was found in *Rag1*^*–/–*^*Smarca4*^*ΔILC3*^ mice. A reduction of ILC3s has been reported during innate colitis, which is correlated with the GM-CSF production and mobilization of ILC3s.^11^ Thus, the trend of reduced ILC3s in *Rag1*^−*/*−^*Smarca4*^*ΔILC3*^ mice could be secondary to spontaneous colitis. Notably, superior expansion of Brg1-deficient ILC3s over controls was observed in both the small and large intestine of the mixed bone marrow chimeric mice, in which no colitis occurred. The above data support a cell-intrinsic effect of Brg1 in suppressing the expansion of both small and large intestinal ILC3s.

We have found that Brg1 is required for the development of NKp46^+^ILC3s and the conversion of NKp46^*−*^ILC3s to NKp46^+^ILC3s in the provision of Notch ligand signal. Previous studies suggest that NKp46^+^ILC3s can be derived from the NKp46^−^CCR6^*–*^T-bet^+^ cells driven by Notch signaling.^23–25^ Consistently, we found the mRNA expression of *Tbx21* and *Rbpj*, a classical transcriptional co-activator of Notch signaling, was significantly decreased in Brg1-deficient NKp46^−^ILC3s.^63^ We detected binding of Brg1 to the locus of *Tbx21* but not *Rbpj* (data not shown) in ILC3s, indicating that RBP-J may be an indirect target for Brg1. RBP-J is curial for the development of NKp46^+^ILC3s and the acquisition of NKp46^+^ILC3s gene features.^23^ However, the molecular mechanism of how RBP-J regulates NKp46^+^ILC3s remains elusive. A previous study has demonstrated that an interaction between Brg1 and Baf60c-containing BAF complex and Notch complex is critical for induction of Nodal at the vertebrate node.^64^ Therefore, it is likely that RBP-J cooperates with Brg1-containing-BAF complex to promote the transcription of *Tbx21* in ILC3s, which is of interest to test in the future.

ILC3-derived GM-CSF has been indicated to be both protective and pathogenic in intestinal inflammation. The expression of GM-CSF needs to be intricately regulated to obtain an optimal dose for the benefit of immune homeostasis. Here, we have demonstrated the Brg1 suppresses the expression GM-CSF from ILC3s by facilitating the H3K27me3 and inhibiting the H3K27ac of the *Csf2* locus. In contrast, Brg1 promotes the expression of *Tbx21* by enhancing the H3K27ac and suppressing H3K27me3 modification. Both positive and negative regulations of gene transcription by BAF have been reported previously in different cell types.^33,34^ Our research has demonstrated that the dual manners of regulation by Brg1 are crucial for homeostatic function of ILC3s.

We have recently reported a synthetic lethality of phosphatase and tensin homolog (PTEN) and Brg1 in prostate cancers.^65^ And Brg1 antagonist has been shown to be effective in treatment of PTEN-deficient prostate cancer.^65^ Since Brg1 is expressed broadly in many types of cells, it is important to determine the function of Brg1 in different subsets of immune cells to avoid unappreciated effects from this potential treatment strategy. ILC3s are gut-resident lymphocytes that have been indicated to play important roles in intestinal infection, autoimmunity, and tumors.^4^ Importantly, intestinal immunity has been associated with systemic diseases through neuronal and metabolic mechanisms.^66,67^ Our work is valuable for understanding the potential risks for therapies targeting Brg1.

## Methods

### Mice

*Rag1*^*−/−*^, *Rorc*^*gfp/gfp*^, *Rorc-cre*, *Cd4-cre*, *Rosa26*^*LSL-YFP/+*^, and B6-Thy1.1 mice were purchased from Jackson laboratory. *Rag2*^*−/−*^*Il2rg*^*−/*−^ mouse was purchased from Taconic Biosciences. *Smarca4*^*flox/flox*^*(Smarca4*^*f/f*^*)* mouse was generated previously.^43^ *Rag1*^*−/−*^*Smarca4*^*f/f*^ mice were crossed to *Rag1*^*−/−*^*Smarca4*^*f/f*^ *Rorc-cre* mice to generate littermate *Rag1*^*−/*−^*Smarca4*^*f/f*^ and *Rag1*^*−/−*^*Smarca4*^ΔILC3^ mice, which were used for experiments in this study. Littermate *Rag1*^*−/−*^*Smarca4*^*f/f*^ and *Rag1*^*−/*−^*Smarca4*^ΔILC3^ mice were kept co-housed after weaning and during experiments. And for cases in which *Rag1*^*−/−*^*Smarca4*^*f/f*^ and *Rag1*^−*/−*^*Smarca4*^ΔILC3^ from different litters were used for experiments, they were gender/age matched and co-housed for more than 2 weeks. For mice of other genotypes used in this study, Brg1-deficient mice and control mice were littermate controlled and co-housed. Both male and female mice were used in this study. All mice were on C57BL/6 background and maintained in specific pathogen free facilities at Shanghai Institute of Nutrition and Health, Chinese Academy of Sciences. All mice experiments were performed in compliance with the guide for the care and use of laboratory animals, approved by the institutional biomedical research ethics committee of the Shanghai Institutes for Biological Sciences, Chinese Academy of Sciences.

### Isolation of intestinal lamina propria lymphocytes (LPLs)

The isolation of intestinal LPLs was performed as previously described.^7^ Briefly, small and large intestines were dissected. Fat tissues were removed. Intestines were dissected longitudinally and subsequently cut into several pieces, followed by washing with cold phosphate-buffered saline (PBS). To remove epithelial cells, the intestines were then incubated successively with 1 mM dithiothreitol (DTT)-PBS once for 10 min at room temperature (RT), and with 30 mM Ethylenediaminetetraacetic acid (EDTA)-PBS twice for 10 min at 37 °C while shaking at 250 rpm. The tissues were then digested with DNase I (Sigma; 150 µg/ml) and collagenase VIII (Sigma; 300 U/ml) in RPMI1640 medium (Thermo Fisher Scientific) at 37 °C in a 5% CO2 incubator for 1.5 h. The digested tissues were homogenized by vigorous shaking and filtered with a 100 µm cell strainer. Mononuclear cells were then harvested from the interphase of an 80 and 40% Percoll (GE Healthcare Life Sciences) gradient after spinning at 2500 rpm for 20 min at RT.

### Cell sorting and flow cytometry

Antibodies used for staining followed by flow cytometry analysis or cell soring were listed in Supplementary Table 1. Dead cells were identified using the LIVE/DEAD Fixable Violet Dead Cell Stain Kit (Thermo Fisher Scientific). Fc receptor blockade was performed using purified α-mouse CD16/CD32 (Supplementary Table 1) before surface staining. The Foxp3 staining kit (Thermo Fisher Scientific) was used for intracellular transcription factor staining. The BD Cytofix/Cytoperm kit was used for intracellular cytokine staining. For preservation of YFP, cells were incubated with 10% formaldehyde solution for 5 min at RT before intracellular transcription factor staining. For Brg1 staining, cells were fixed using the BD Cytofix/Cytoperm kit for 20 min after surface staining, followed by permeabilization using ice cold methanol. Flow cytometry data were collected using the Gallios flow cytometer (Beckman Coulter) and analyzed by FlowJo software (Tree Star Inc.). For detection of cytokine production, intestinal LPLs were treated with IL-1β (10 ng/ml), IL-23 (10 ng/ml), TL1A (10 ng/ml) or PBS control for 12 h, or with 50 ng/ml PMA (Sigma), and 500 ng/ml ionomycin (Sigma) for 3 h. And 2 μg/ml Brefeldin A (Sigma) was added to the culture for the last 3 h before cells were harvested for analysis. The lineage markers (Lin) were defined as CD3ε, B220, CD11b, and CD11c in all experiments. ILC3s were sorted as Thy1.2^high^CD45^low^ cells from *Rag1*^−*/−*^ mice or as Lin^−^RORγt-GFP^+^ cells from *Rorc*^*gfp/+*^ mice using BD FACSAria III or MoFlo Astrios (Beckman Coulter).^68^

### Cell culture medium

Reagents used for making complete medium for cell culture were from Thermo Fisher Scientific, except for IMDM and β-Mercaptoethanol (β-ME) were from Sigma-Aldrich. IMDM complete medium was prepared using IMDM medium supplemented with 10% fetal bovine serum, 2 mM L-glutamine, 1 mM sodium pyruvate, 1 mM HEPES, 50 µM β-ME, and 1% penicillin/streptomycin. α-MEM complete medium was prepared using MEM ALPHA supplemented with 20% fetal bovine serum, 1 mM HEPES, 50 µM β-ME, 100 U/ml penicillin, and 100 µg/ml streptomycin.

### ILC3 in vitro differentiation

5000–10,000 of OP9-GFP or OP9-DLL4 cells, generated previously,^69^ were plated into 96-well flat-bottom plate as feeder cells and cultured for overnight. NKp46^*−*^ILC3s were purified from small intestinal LPLs of *Rag1*^*−/−*^*Smarca4*^*f/f*^*Rorc-cre* or *Rag1*^−*/−*^*Smarca4*^*f/f*^ mice. Feeder cells were treated with mitomycin-C (50 µg/ml) for 30 min and cultured with 10,000–50,000 NKp46^−^ILC3s in α-MEM complete medium in the presence of 20 ng/ml Flt3L (Peprotech), 10 ng/ml IL-7 (Peprotech), and 10 ng/ml SCF (Biolegend) for 7 days before analysis.

### In vivo antibody treatment

α-Thy1.2 (30H12), α-GM-CSF (MP1–22E9), and α-IL17F (MM17F8F5.1A9) antibodies were purchased from BioXcell. Rat IgG was purchased from Sango Biotech. *Rag1*^*−/−*^*Smarca4*^*f/f*^*Rorc-cre* and *Rag1*^*−/*−^*Smarca4*^*f/f*^ mice were administered intraperitoneally with 250 µg per mouse of indicated antibodies or control IgG every 3 days continuously for 14 days before analysis.

### Construction of mixed bone marrow chimeric mice

Bone marrow cells from Thy1.2/1.2*Rag1*^*−/−*^*Smarca4*^*f/f*^ and Thy1.1/1.2*Rag1*^*−/*–^*Smarca4*^*ΔILC3*^ mice, or alternatively from Thy1.1/1.2*Rag1*^*−/*−^*Smarca4*^*f/f*^ and Thy1.2/1.2*Rag1*^*−/*−^*Smarca4*^*ΔILC3*^, or alternatively Thy1.1/1.2*Rag1*^*−/−*^*Smarca4*^*f/f*^ and Thy1.1/1.1*Rag1*^*−/−*^*Smarca4*^*ΔILC3*^, were mixed at 1:1 ratio (2.5 × 10^6^ cells from each donor) and transferred to sublethally (550 rads) irradiated *Rag2*^*−/*−^*Il2rg*^−*/−*^ mice. Mice were sacrificed for analyses 6–8 weeks later.

### Histological assessment of intestinal inflammation

Colons were dissected and fixed in 4% paraformaldehyde solution for at least 72 h and intestinal Swiss rolls were embedded in paraffin blocks. 4–7 µm of tissue sections were stained with H&E, and the severity of disease was scored according to a previously described criterion in a blinded fashion.^70^ In brief, four parameters were assessed and scored as 0–3: epithelial hyperplasia and goblet cell depletion, leukocyte infiltration in the lamina propria, area of tissue affected, and markers of severe inflammation such as submucosal inflammation. A sum score for the four parameters was calculated, and the histological score for one section was the average of sum scores from four quadrants scopes of each section.

### Detection of mRNA by real-time RT-PCR

RNA was isolated with Trizol reagent (Invitrogen). cDNA was synthesized using a GoScript™ Reverse Transcription kit (Promega). Real-time PCR was performed using SYBR Green Master Mix reagents (Roche) and primer mixtures (Supplementary Table 2) were used for the real-time PCR. Reactions were run with the Chromatin Immunoprecipitation (ChIP) coupled with quantitative PCR. The results were displayed as relative expression values normalized to β-actin.

### RNA-seq analysis

Bone marrow cells from Thy1.2/1.2*Rag1*^*−/*−^*Smarca4*^*f/f*^ and Thy1.1/1.2*Rag1*^*−/*−^*Smarca4*^*ΔILC3*^ mice were mixed at 1:1 ratio (2.5 × 10^6^ cells from each donor) and transferred to sublethally (550 rads) irradiated *Rag2*^*−/*−^*Il2rg*^*−/*−^ mice. Recipient mice were then sacrificed for 6 weeks later. ILC3s from the Thy1.2/1.2*Rag1*^−*/*−^*Smarca4*^*f/f*^ origin (Thy1.2^high^Thy1.1^*−*^CD45^low^) and the Thy1.1/1.2*Rag1*^−*/*−^*Smarca4*^*f/f*^*Rorc-cre* origin (Thy1.2^high^Thy1.1^+^CD45^low^) were sorted from the small intestinal LPL by flow cytometry and lysed in Trizol (Invitrogen). Total RNA was extracted. Biological duplicates were generated for each group. Construction of the cDNA library and 50 bp single end transcriptome sequencing were performed by BGI Genomics, BGI-Shenzhen, as following. First-strand cDNA was generated by SuperScript III reverse transcriptase (Thermo Fisher Scientific). Free primers were removed using Exonuclease I (New England Biolabs). 3′ poly (A) tailing was done by adding terminal deoxynucleotidyl transferase (Thermo Fisher Scientific). Then, the second-strand cDNA was synthesized and the cDNA was amplified by PCR. PCR products were purified with Ampure XP Beads (Beckman Coulter). After that, second round of PCR was performed and PCR products were fragmented by a sonicator (Covaris). End repaired was performed and A-tailing was added. Adaptor ligation was done and the cDNA was further amplified by PCR and purified with Ampure XP Beads. Fragment sizes were checked using Agilent 2100 bioanalyzer instrument (Agilent DNA 1000 Reagents) and the library was quantified by real-time quantitative PCR (Q-PCR) (TaqMan Probe). The qualified libraries were amplified on cBot to generate the cluster on the flowcell (TruSeq PE Cluster Kit V3–cBot–HS, Illumina). And the amplified flowcell was sequenced single end on the HiSeq 2000 System (TruSeq SBS KIT-HS V3, Illumina) with reading length of 90 bp. Reads were mapped to Mouse Genome Assembly GRCm38.p5 by STAR v2.5. Gene and isoform expression quantification was called by RSEM v1.2 with default parameters on GENCODE mouse M16 gene annotation file. Differential expression analysis was performed by Bioconductor package edgeR v3.18.1. Significantly changed genes were chosen according to two criteria: (1) significance level FDR < 0.05; (2) expression level average FPKM values bigger than 1 in either treatment or control groups. The fastq files were stored in the Gene Expression Omnibus public database (accession no. GSE133853). Significantly changed genes used for gene ontology enrichment analysis using the website of DAVID Bioinformatics Resources 6.8 (https://david.ncifcrf.gov/), were filtered with the following criteria: (1) significance level *p* < 0.05, FDR < 0.4; (2) expression level average FPKM values bigger than 5 in either group. (3) Fold change of mean expression between KO and WT group is more than 2^0.5^. Normalized heatmap was based on the standard score (*Z* score) and generated with GraphPad Prism program. The standard score of a raw score × is $Z \,=\, \frac{{x \,-\, \mu }}{\sigma }$. *μ* is the mean of the FPKM value of each sample and σ is the standard deviation of the FPKM value of each sample.

### ATAC-seq analysis and integrated analysis with RNA-seq

Biological triplicates of 50,000 ILC3s (Thy1.2^high^CD45^low^ cells) sorted by flow cytometry from the small intestine of 7–9-week-old *Rag1*^*−/−*^*Smarca4*^*f/f*^ or *Rag1*^*−/*−^*Smarca4*^*ΔILC3*^ mice were subjected to ATAC-seq analysis. Library preparation was performed using TruePrepTM DNA Library Prep Kit V2 for Illumina® kit from Vanzyme (Nanjing, China). 1.2 × Agencourt AMPure XP beads (Beckman Coulter) were used to purify libraries for sequencing. One hundred fifty paired-end sequencing was performed with Navoseq 6000.

ATAC-seq data analyses were performed by DIATRE Biotechnology, Shanghai, China. Raw sequence reads were initially processed by FastQC for quality control, and then adapter sequences and poor quality reads were removed. Quality filtered reads were then mapped to mouse genome (mm10) using Bowtie2, and only uniquely mapped reads were kept. Sam files were converted to Bam format using Samtools. Peak calling was done using MACS2 with an initial threshold *q-*value of 0.01 as cutoff. Differentially expressed ATAC-seq peaks were identified by firstly quantifying peak signal using bedtools multicov, and then using DESeq2 for differential analysis. Peaks with differential expression of more than 1.5-fold were designated as specific OCRs for wild-type ILC3s (WT-ILC3-OCRs) or for Brg1-deficient ILC3 (Brg1-KO-ILC3-OCRs). Visualization of read count data was performed by converting raw bam files to bigwig files using IGV tools. The accession number for ATAC-seq data is GEO: GSE148078. For integrated analysis of ATAC-seq and RNA-seq data (done in Fig. 5), the correlation of mRNA expression (Brg1-deficient ILC3 over WT ILC3) with chromatin accessibility was performed using Log2 fold change of mRNA expression with average Log2 fold change of accessibility of peaks in promoter, intragenic (5′UTR + 3′UTR + exon + intron) or intergenic regions annotated by Homer2 (Brg1-deficient ILC3 over WT ILC3). Average folder change of accessibility was calculated by fold in accessibility of all observed peaks divided by the number of observed peaks.

### Chromatin immunoprecipitation (ChIP)-quantitative PCR (qPCR) assays

FACS-sorted ILC3s were fixed by 1% formaldehyde (Sigma) and fragmented by sonication to 100–500 bp. α-H3K27ac (Abcam, ab4729), α-H3K27me3 (Abcam, ab222481), α-Brg1 (Abcam, ab215998) antibody or Rabbit IgG (Abcam, ab171870) were then used for immunoprecipitation. After being washed and reverse crosslinked, the pull-down DNA, as well as the input DNA, was purified by ethanol precipitation and quantified using real-time PCR. Primers used for ChIP-qPCR were listed in Supplementary Table 2.

### Statistical analysis

Unless otherwise noted, Kolmogorov–Smirnov test was performed for normality tests. Statistical analysis was then performed using the unpaired Student’s *t* test as parametric tests or Mann–Whitney test as nonparametric tests on individual biological samples using GraphPad Prism program. Data from these experiments are presented as mean values ± SEM. *p < 0.05; **p < 0.01; ***p < 0.001; ****p < 0.0001.

## Supplementary information

Supplementary Information

Supplementary Data 1

Supplementary Data 2

Supplementary Data 3
