# Correction: Brg1 restrains the pro-inflammatory properties of ILC3s and modulates intestinal immunity

**DOI:** 10.1038/s41385-020-0324-4

**Published:** 2020-07-23

**Authors:** Xinyi Qi, Jinxin Qiu, Jiali Chang, Yan Ji, Qi Yang, Guoliang Cui, Liming Sun, Qian Chai, Jun Qin, Ju Qiu

**Affiliations:** 1 CAS Key Laboratory of Tissue Microenvironment and Tumor, Shanghai Institutes for Biological Sciences (SIBS), Chinese Academy of Sciences (CAS), Shanghai Jiao Tong University School of Medicine (SJTUSM), Shanghai, 200031 China; 2 CAS Key Laboratory of Tissue Microenvironment and Tumor, Shanghai Institute of Nutrition and Health, Shanghai Institutes for Biological Sciences, Chinese Academy of Sciences, University of Chinese Academy of Sciences, Shanghai, 200031 China; 3 Department of Immunology and Microbial Disease, Albany Medical College, Albany, NY 12208 USA; 4 T Cell Metabolism Group (D140), German Cancer Research Center (DKFZ), Im Neuenheimer Feld 280, 69120 Heidelberg, Germany; 5 Faculty of Biosciences, Heidelberg University, 69120 Heidelberg, Germany; 6 State Key Laboratory of Cell Biology, CAS Center for Excellence in Molecular Cell Science, Shanghai Institute of Biochemistry and Cell Biology, Chinese Academy of Sciences, University of Chinese Academy of Sciences, Shanghai, 200031 China; 7 Key Laboratory of Infection and Immunity, Institute of Biophysics, Chinese Academy of Sciences, 100101 Beijing, China

Correction to: *Mucosal Immunology* 10.1038/s41385-020-0317-3, published online 1 July 2020

In the original version of this Article, the affiliations of both authors, Liming Sun and Qian Chai, were inadvertently switched.

The correct affiliation for Liming Sun should be: State Key Laboratory of Cell Biology, CAS Center for Excellence in Molecular Cell Science, Shanghai Institute of Biochemistry and Cell Biology, Chinese Academy of Sciences, University of Chinese Academy of Sciences, 200031, Shanghai, China.

And the correct affiliation for Qian Chai should be: Key Laboratory of Infection and Immunity, Institute of Biophysics, Chinese Academy of Sciences, 100101, Beijing China.

This has now been corrected in both the PDF and HTML versions of the Article.

