# Supplementary material for: Brg1 restrains the pro-inflammatory properties of ILC3s and modulates intestinal immunity
Source: Mucosal Immunol. 2020 Jul 1;14(1):38–52. doi: 10.1038/s41385-020-0317-3 (PMC7790751; doi:10.1038/s41385-020-0317-3)
Supplement: Supplementary file 1 — Supplementary Information [file 41385_2020_317_MOESM1_ESM.pdf]

Supplementary Information

Qi et. al.

Figure S1

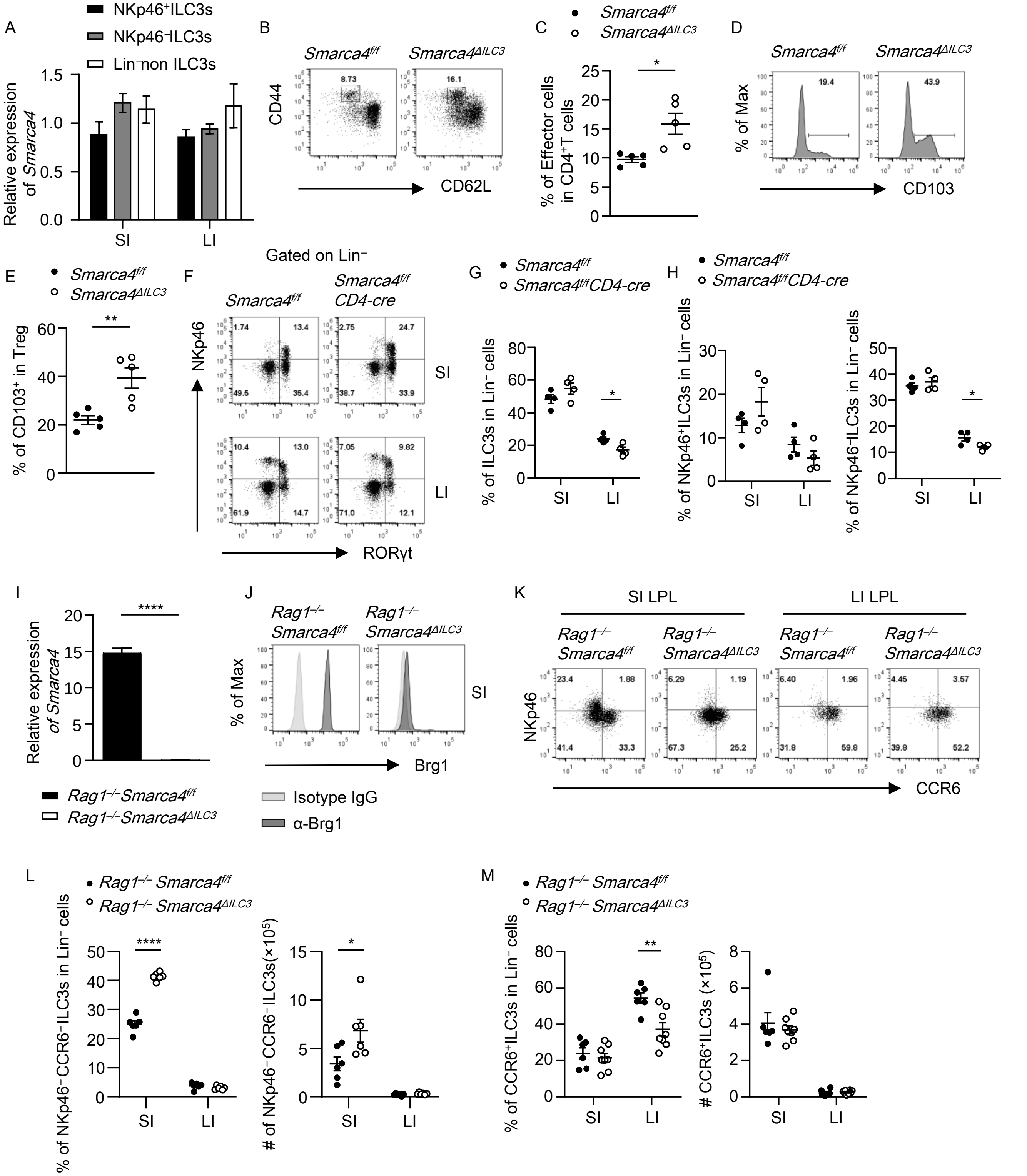
A
NKp46+ILC3s
NKp46−ILC3s
Lin−non ILC3s
Relative expression of Smarca4
SI
LI
B
Smarca4f/f
Smarca4ΔILC3
CD44
CD62L
8.73
16.1
C
% of Effector cells in CD4+T cells
D
% of Max
CD103
19.4
43.9
E
% of CD103+ in Treg
Gated on Lin−
F
Smarca4f/f CD4-cre
NKp46
RORγt
G
Smarca4f/fCD4-cre
% of ILC3s in Lin− cells
H
% of NKp46+ILC3s in Lin− cells
% of NKp46−ILC3s in Lin− cells
I
Rag1−/−Smarca4f/f
Rag1−/−Smarca4ΔILC3
J
Isotype IgG
α-Brg1
Brg1
K
SI LPL
LI LPL
CCR6
L
% of NKp46−CCR6−ILC3s in Lin− cells
# of NKp46−CCR6−ILC3s(×10^5)
M
% of CCR6+ILC3s in Lin− cells
# CCR6+ILC3s (×10^5)

Figure S2

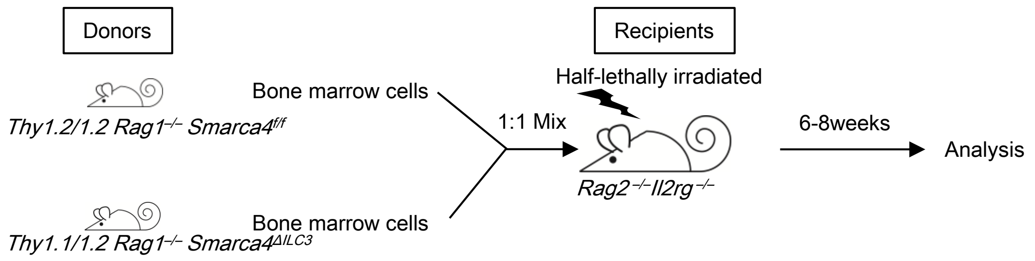
Donors
Recipients
Half-lethally irradiated
Bone marrow cells
Thy1.2/1.2 Rag1−/− Smarca4f/f
1:1 Mix
6-8weeks
Analysis
Rag2−/−Il2rg−/−
Bone marrow cells
Thy1.1/1.2 Rag1−/− Smarca4ΔILC3

Figure S3

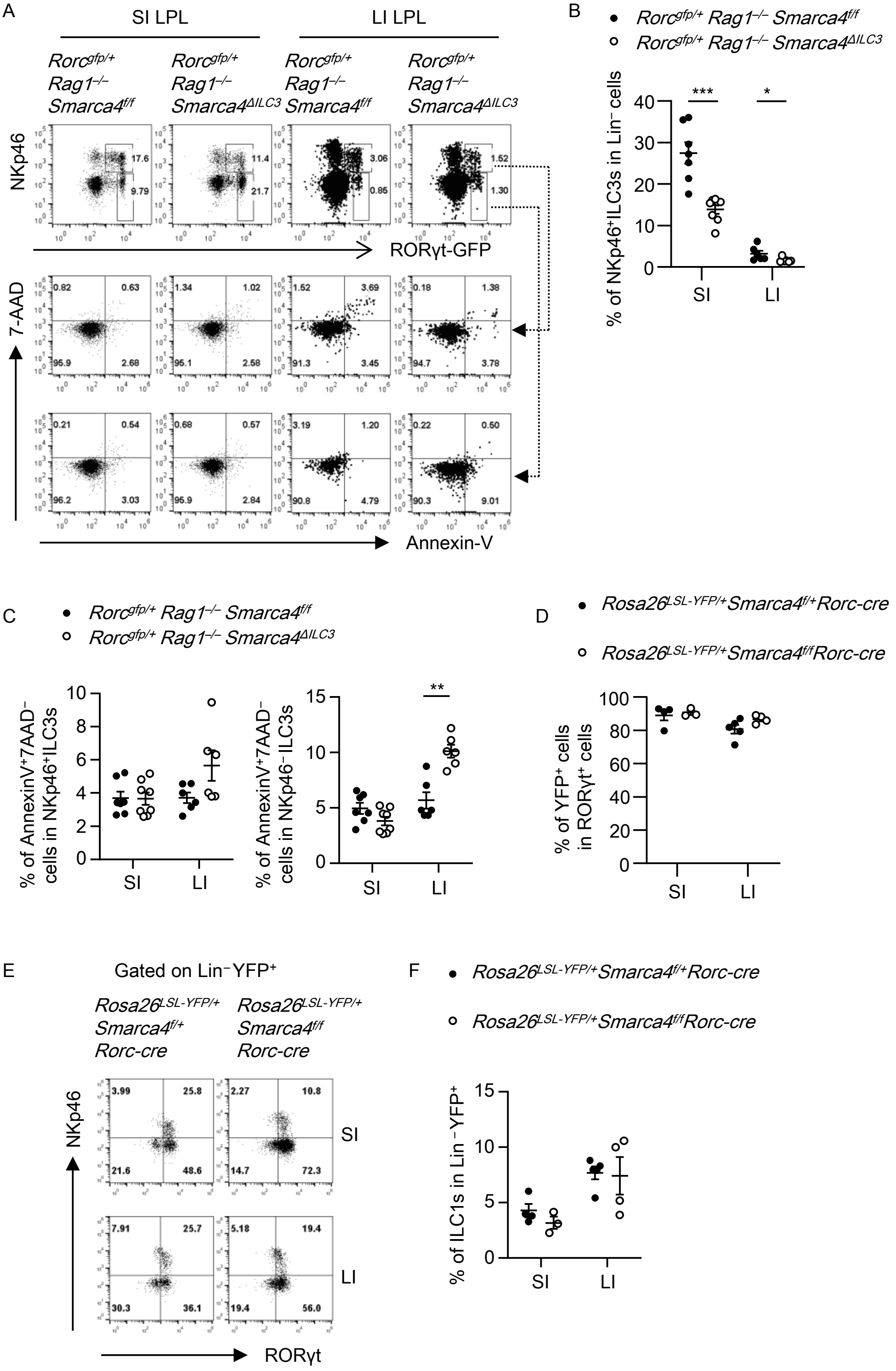
A
SI LPL
LI LPL
Rorcgfp/+ Rag1–/– Smarca4f/f
Rorcgfp/+ Rag1–/– Smarca4ΔILC3
NKp46
RORγt-GFP
7-AAD
Annexin-V
17.6
9.79
11.4
21.7
3.06
0.85
1.52
1.30
0.82
0.63
95.9
2.68
1.34
1.02
95.1
2.58
1.52
3.69
91.3
3.45
0.18
1.38
94.7
3.78
0.21
0.54
96.2
3.03
0.68
0.57
95.9
2.84
3.19
1.20
90.8
4.79
0.22
0.50
90.3
9.01
B
Rorcgfp/+ Rag1–/– Smarca4f/f
Rorcgfp/+ Rag1–/– Smarca4ΔILC3
% of NKp46+ILC3s in Lin− cells
***
*
SI
LI
C
% of AnnexinV+7AAD− cells in NKp46+ILC3s
% of AnnexinV+7AAD− cells in NKp46−ILC3s
**
D
Rosa26LSL-YFP/+Smarca4f/+Rorc-cre
Rosa26LSL-YFP/+Smarca4f/fRorc-cre
% of YFP+ cells in RORγt+ cells
E
Gated on Lin−YFP+
Rosa26LSL-YFP/+ Smarca4f/+ Rorc-cre
Rosa26LSL-YFP/+ Smarca4f/f Rorc-cre
NKp46
RORγt
3.99
25.8
21.6
48.6
2.27
10.8
14.7
72.3
7.91
25.7
30.3
36.1
5.18
19.4
19.4
56.0
F
% of ILC1s in Lin−YFP+

Figure S4

A

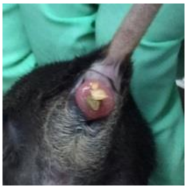

Rag1$^{-/-}$ *Smarca4*$^{\Delta ILC3}$

	Diarrhea	Rectal prolapse	Rectal bleeding
Rag1$^{-/-}$ *Smarca4*$^{\Delta ILC3}$	77.78%	33.33%	7.41%

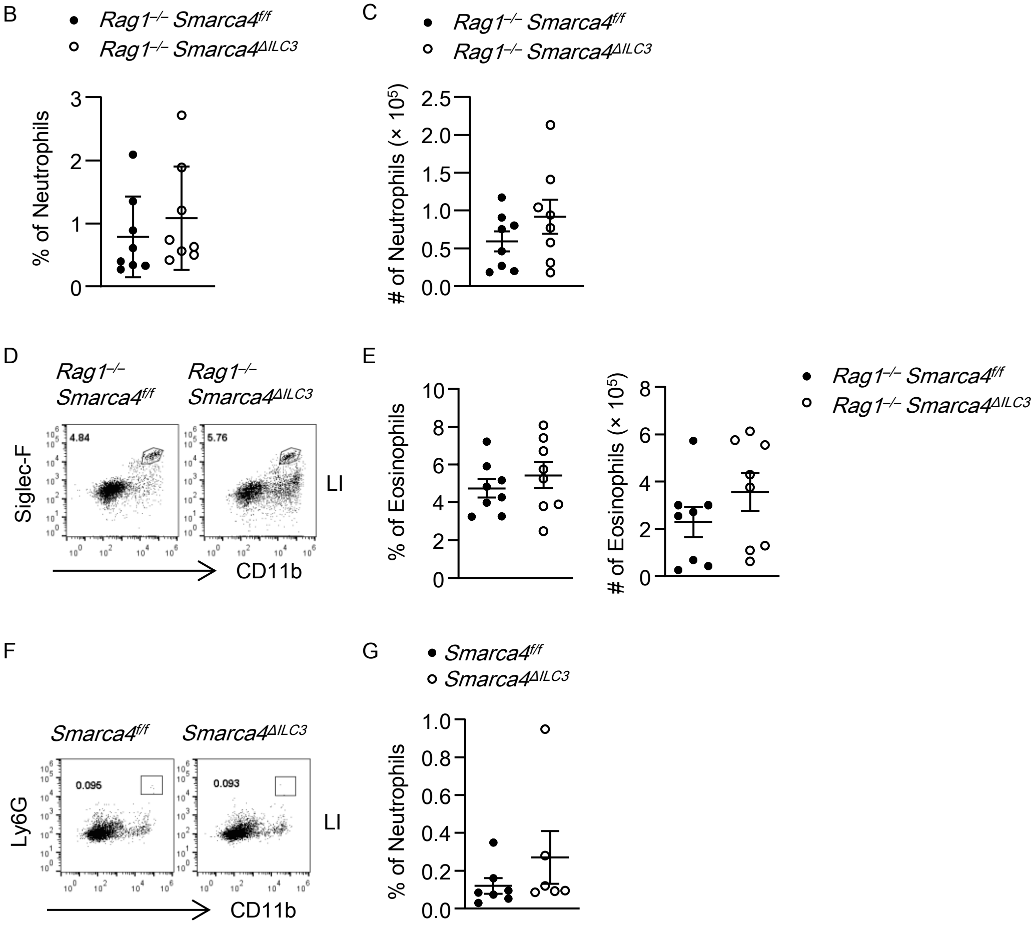

Figure S5

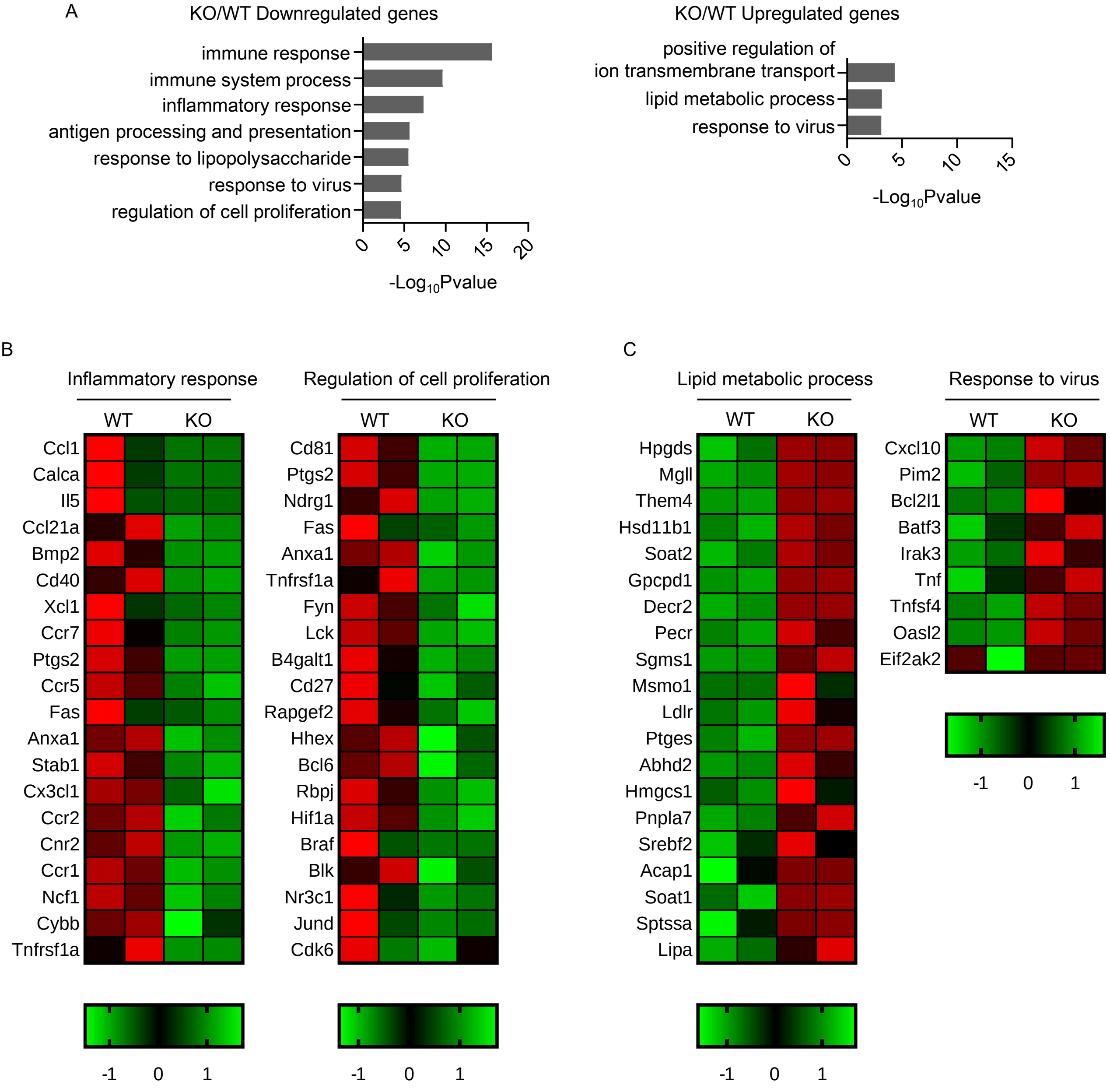

A
KO/WT Downregulated genes
immune response
immune system process
inflammatory response
antigen processing and presentation
response to lipopolysaccharide
response to virus
regulation of cell proliferation
0 5 10 15 20
-Log10Pvalue
KO/WT Upregulated genes
positive regulation of ion transmembrane transport
lipid metabolic process
response to virus
0 5 10 15
-Log10Pvalue
B
Inflammatory response
WT KO
Ccl1 Calca Il5 Ccl21a Bmp2 Cd40 Xcl1 Ccr7 Ptgs2 Ccr5 Fas Anxa1 Stab1 Cx3cl1 Ccr2 Cnr2 Ccr1 Ncf1 Cybb Tnfrsf1a
-1 0 1
Regulation of cell proliferation
WT KO
Cd81 Ptgs2 Ndrg1 Fas Anxa1 Tnfrsf1a Fyn Lck B4galt1 Cd27 Rapgef2 Hhex Bcl6 Rbpj Hif1a Braf Blk Nr3c1 Jund Cdk6
-1 0 1
C
Lipid metabolic process
WT KO
Hpgds Mgll Them4 Hsd11b1 Soat2 Gpcpd1 Decr2 Pecr Sgms1 Msmo1 Ldlr Ptges Abhd2 Hmgcs1 Pnpla7 Srebf2 Acap1 Soat1 Sptssa Lipa
-1 0 1
Response to virus
WT KO
Cxcl10 Pim2 Bcl2l1 Batf3 Irak3 Tnf Tnfsf4 Oasl2 Eif2ak2
-1 0 1

Figure S6

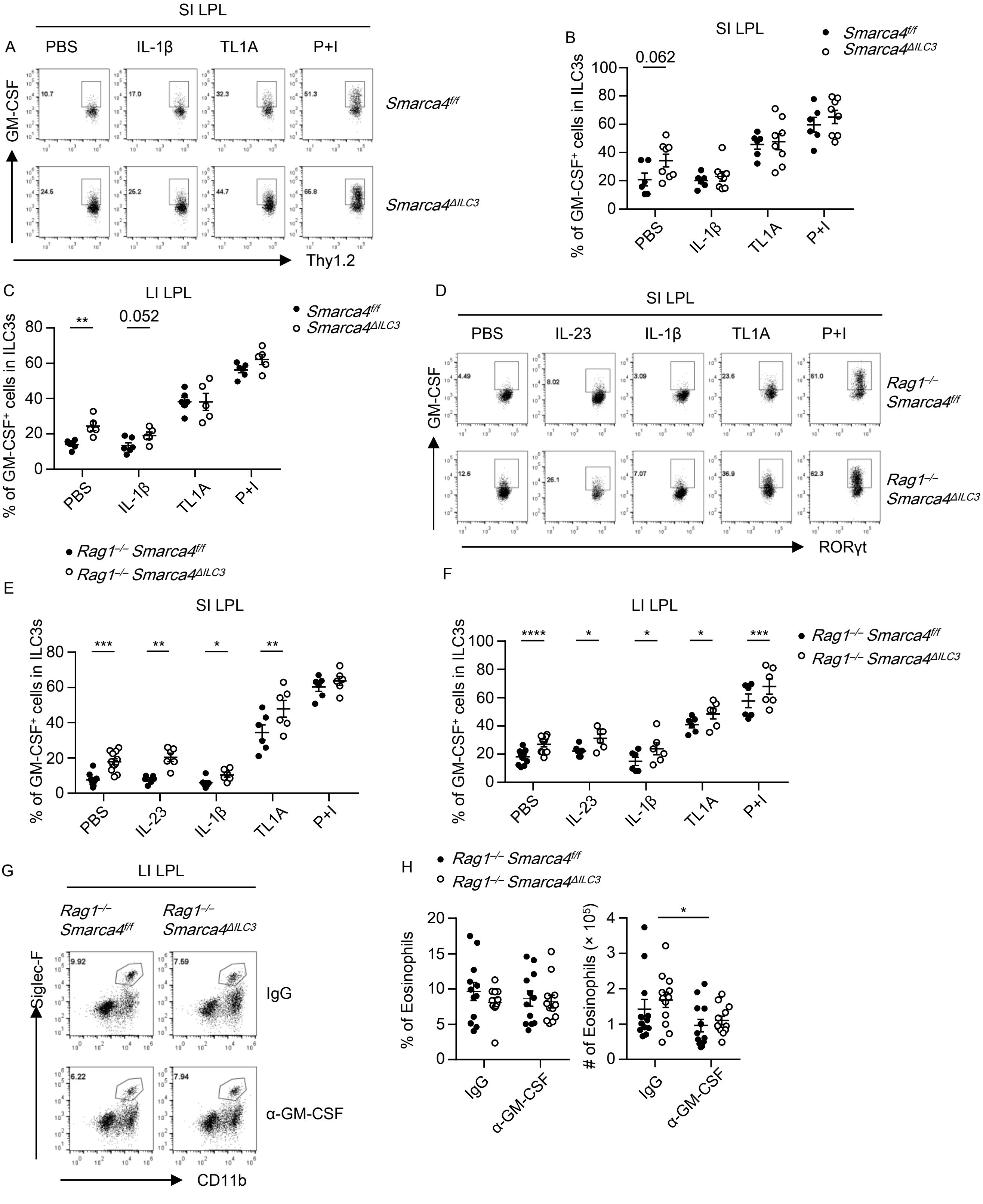

A
SI LPL
PBS
IL-1β
TL1A
P+I
GM-CSF
Thy1.2
10.7
17.0
32.3
51.3
24.5
25.2
44.7
65.8
Smarca4f/f
Smarca4ΔILC3
B
SI LPL
0.062
% of GM-CSF+ cells in ILC3s
C
LI LPL
**
0.052
D
SI LPL
IL-23
RORγt
4.49
8.02
3.09
23.6
61.0
12.6
26.1
7.07
36.9
62.3
Rag1–/– Smarca4f/f
Rag1–/– Smarca4ΔILC3
E
***
*
F
****
G
LI LPL
Siglec-F
CD11b
9.92
7.59
6.22
7.94
IgG
α-GM-CSF
H
% of Eosinophils
# of Eosinophils (× 10^5)

Figure S7

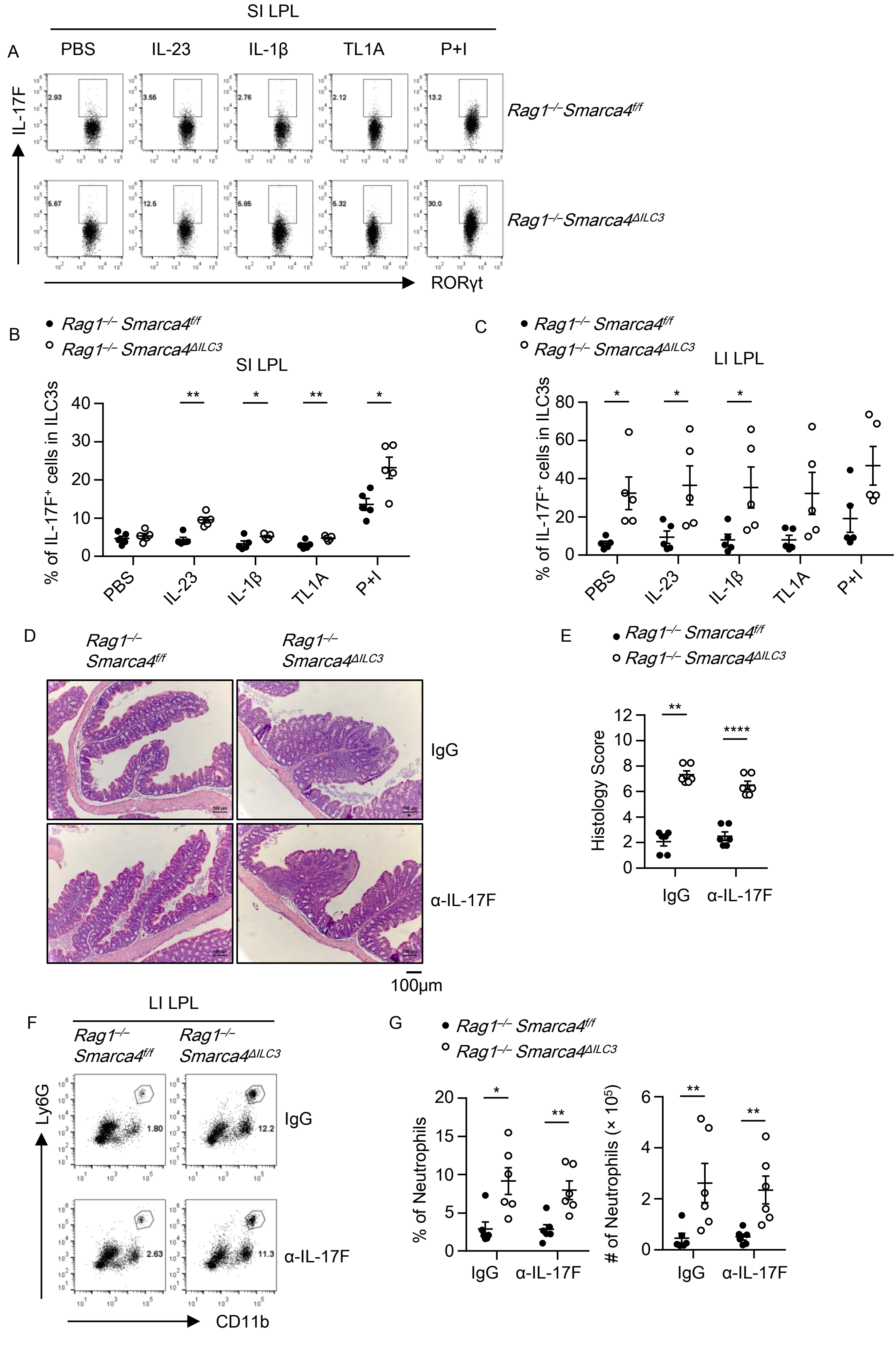

SI LPL
A
PBS
IL-23
IL-1β
TL1A
P+I
IL-17F
RORγt
Rag1–/–Smarca4f/f
Rag1–/–Smarca4ΔILC3
B
SI LPL
% of IL-17F+ cells in ILC3s
C
LI LPL
D
IgG
α-IL-17F
100μm
E
Histology Score
F
LI LPL
Ly6G
CD11b
G
% of Neutrophils
# of Neutrophils (× 10^5)

Figure S8

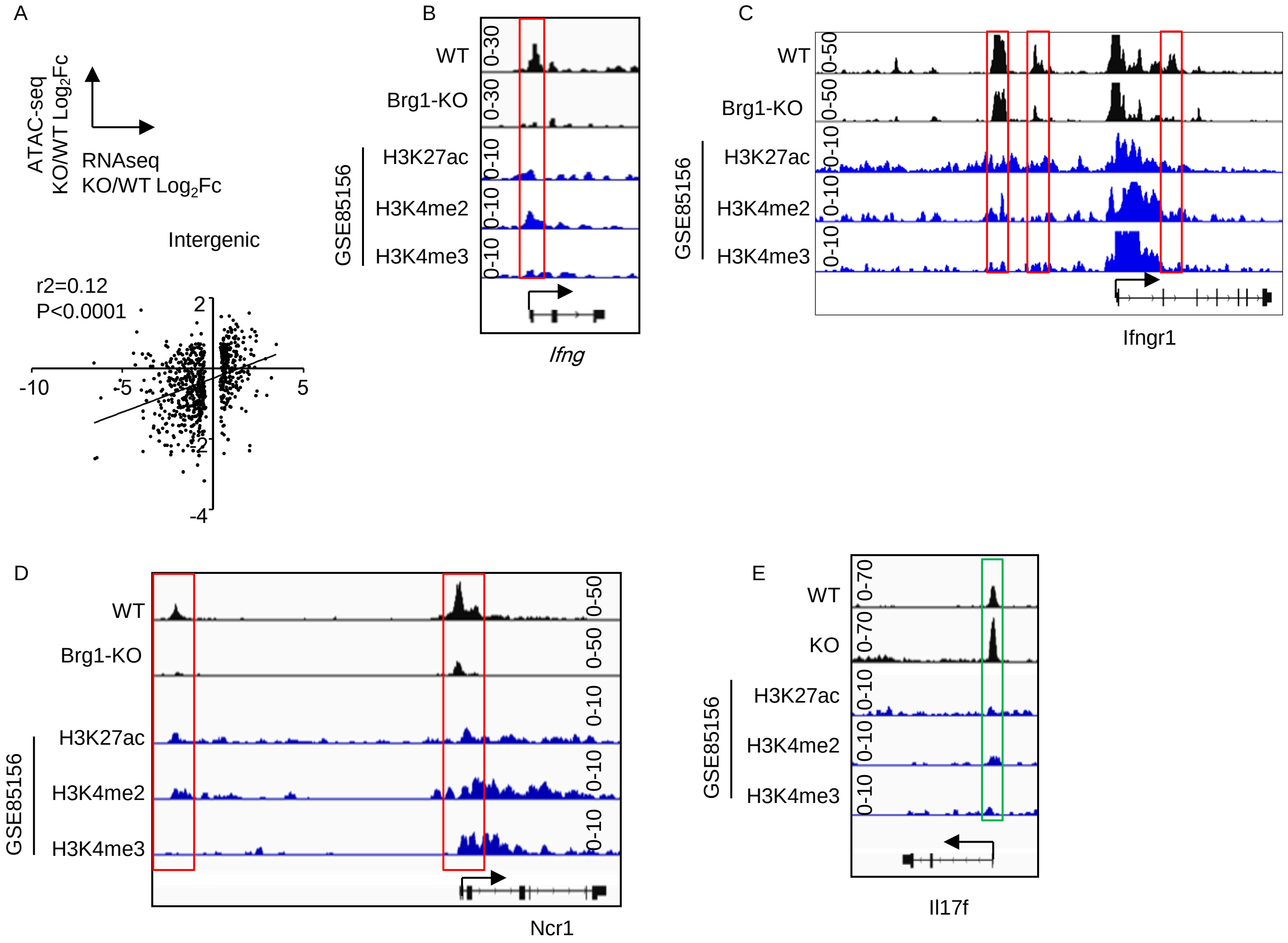
A
ATAC-seq KO/WT Log2Fc
RNAseq KO/WT Log2Fc
Intergenic
r2=0.12
P<0.0001
2
-2
-4
-10
-5
5
B
WT
Brg1-KO
GSE85156
H3K27ac
H3K4me2
H3K4me3
0-30
0-10
Ifng
C
WT
Brg1-KO
H3K27ac
H3K4me2
H3K4me3
0-50
0-10
Ifngr1
D
WT
Brg1-KO
H3K27ac
H3K4me2
H3K4me3
0-50
0-10
Ncr1
E
WT
KO
H3K27ac
H3K4me2
H3K4me3
0-70
0-10
Il17f

Supplementary Figure 1 Brg1 is expressed by ILC3s and deletion of Brg1 in T cells has no effect on development of NKp46$^+$ILC3s

(A) Relative expression of *Smarca4* in NKp46$^+$ILC3s (Lin$^-$RORγt-GFP$^+$NKp46$^+$), NKp46$^-$ ILC3s (Lin$^-$RORγt-GFP$^+$NKp46$^-$) and other ILCs (Lin$^-$RORγt-GFP$^-$) sorted from small (SI) and large (LI) intestinal lamina propria lymphocytes (LPLs) of the *Rorc*$^{gfp/+}$ mice (n=3; Representative of 2 experiments). (B-E) Splenocytes were isolated from *Smarca4*$^{f/f}$ or *Smarca4*$^{f/f}$*Rorc-cre (Smarca4*$^{\Delta ILC3}$*)* mice (n=5; Representative of 2 experiments). (B) Representative flow cytometry plots for effector CD4$^+$T (CD44^{hi}CD62L^{low}) cells gated on CD3$^+$CD4$^+$ cells. (C) Frequencies of effector CD4$^+$T cells in CD3$^+$CD4$^+$ cells. (D) Representative flow cytometry plots showing CD103 expression gated on CD3$^+$CD4$^+$Foxp3$^+$ regulatory T cells (Treg cells). (E) Frequencies of CD103$^+$ cells in Treg cells. (F-H) SI and LI LPLs were isolated from *Smarca4*$^{f/f}$ and *Smarca4*$^{f/f}$*CD4-cre* mice (n=4; Representative of 2 experiments). (F) Representative flow cytometry plots for NKp46$^+$ILC3s (Lin$^-$ RORγt$^+$NKp46$^+$) and NKp46$^-$ILC3s (Lin$^-$RORγt$^+$NKp46$^-$) gated on Lin$^-$ cells. (G) Frequencies of ILC3s (Lin$^-$RORγt$^+$) in Lin$^-$ cells. (H) Frequencies of NKp46$^+$ILC3s and NKp46$^-$ILC3s in Lin$^-$ cells. (I) Relative *Smarca4* mRNA expression in ILC3s (CD45^{low}Thy1.2^{high}) isolated from SI LPLs of *Rag1*$^{-/-}$*Smarca4*$^{f/f}$ and *Rag1*$^{-/-}$*Smarca4*$^{f/f}$*Rorc-cre (Rag1*$^{-/-}$*Smarca4*$^{\Delta ILC3}$*)* mice (n=6; Representative of 2 experiments). (J) Representative flow cytometry plots showing Brg1 expression gated on ILC3s (Lin$^-$RORγt$^+$) from SI LPLs of *Rag1*$^{-/-}$*Smarca4*$^{f/f}$ and *Rag1*$^{-/-}$*Smarca4*$^{\Delta ILC3}$ mice. (K-M) SI and LI LPLs were isolated from *Rag1*$^{-/-}$*Smarca4*$^{f/f}$ and *Rag1*$^{-/-}$*Smarca4*$^{\Delta ILC3}$ mice (n=6-8; Representative of 3 experiments). (K) Representative flow cytometry plots for NKp46$^+$ILC3s (Lin$^-$

RORγt$^+$NKp46$^+$), NKp46$^-$CCR6$^-$ILC3s (Lin$^-$RORγt$^+$NKp46$^-$CCR6$^-$) and CCR6$^+$ILC3s (Lin$^-$RORγt$^+$CCR6$^+$) gated on ILC3s. (L) Frequencies and total numbers of NKp46$^-$CCR6$^-$ILC3s in Lin$^-$ cells (calculated by frequencies of NKp46$^-$CCR6$^-$ILC3s in ILC3s multiplying frequencies of ILC3s in Lin$^-$ cells). (M) Frequencies and total numbers of CCR6$^+$ILC3s in Lin$^-$ cells (calculated by CCR6$^+$ILC3s in ILC3s multiplying frequencies of ILC3s in Lin$^-$ cells). (A-M) Data are represented as means ± SEM. Error bars show SEM. *P<0.05, Lin$^-$, CD3ε$^-$B220$^-$CD11b$^-$CD11c$^-$.

Supplementary Figure 2 Schematic chart showing the construction of bone marrow chimeric mouse

Bone marrow cells from Thy1.2/1.2*Rag1*$^{-/-}$*Smarca4*$^{f/f}$ and Thy1.1/1.2*Rag1*$^{-/-}$*Smarca4*$^{\Delta ILC3}$ mice, or alternatively from Thy1.1/1.2*Rag1*$^{-/-}$*Smarca4*$^{f/f}$ and Thy1.2/1.2*Rag1*$^{-/-}$*Smarca4*$^{\Delta ILC3}$, or alternatively Thy1.1/1.2*Rag1*$^{-/-}$*Smarca4*$^{f/f}$ and Thy1.1/1.1*Rag1*$^{-/-}$*Smarca4*$^{\Delta ILC3}$, were mixed at 1:1 ratio (2.5×10^6 cells from each donor) and transferred to sub-lethally irradiated *Rag2*$^{-/-}$*Il2rg*$^{-/-}$ mice. Mice were sacrificed for analyses 6-8 weeks later.

Supplementary Figure 3 Reduction of NKp46$^+$ILC3s in the absence of Brg1 is not due to apoptosis or fate conversion

(A-C) SI and LI LPLs were isolated from *Rag1*$^{-/-}$*Smarca4*$^{f/f}$*Rorc*$^{gfp/+}$ and *Rag1*$^{-/-}$*Smarca4*$^{\Delta ILC3}$*Rorc*$^{gfp/+}$ mice (n=6; Representative of 3 experiments). (A) Representative flow cytometry plots showing gating strategy for analysis of cell apoptosis in NKp46$^+$ILC3s (Lin$^-$RORγt-GFP$^+$NKp46$^+$) and NKp46$^-$ILC3s (Lin$^-$RORγt-GFP$^+$NKp46$^-$) based on Annexin-V

and 7-AAD staining. (B) Frequencies of NKp46^{+}ILC3s in Lin^{-} cells. (C) Frequencies of apoptotic cells (Annexin-V^{+}7-AAD^{-}) in NKp46^{+}ILC3s and NKp46^{-}ILC3s. (D-F) SI and LI LPLs were isolated from *Rosa26*$^{LSL\text{-}YFP/+}$*Smarca4*$^{f/+}$*Rorc-cre* or *Rosa26*$^{LSL\text{-}YFP/+}$*Smarca4*$^{f/f}$*Rorc-cre* adult mice (n=3-5, Representative of 3 experiments). (D) Representative flow cytometry plots for FM^{+}ILC1s (Lin^{-}YFP^{+}RORγt^{-}NKp46^{+}) gated on Lin^{-}YFP^{+} cells. (E) Penetrance for YFP marking of the RORγt^{+} cells. (F) Frequencies of FM^{+}ILC1s (Lin^{-}YFP^{+}RORγt^{-}NKp46^{+}) in Lin^{-}YFP^{+} cells. (A-F) Data are represented as means ± SEM. Error bars show SEM. **P<0.01. FM, fate mapping; Lin^{-}, CD3ε^{-}B220^{-}CD11b^{-}CD11c^{-}.

Supplementary Figure 4 No signs of inflammation in the small intestine and no increased infiltration of eosinophils in the large intestine of *Rag1*$^{-/-}$*Smarca4*$^{\Delta ILC3}$ mice

(A) Representative image showing rectal prolapse in *Rag1*$^{-/-}$*Smarca4*$^{\Delta ILC3}$ mice and incidences of diarrhea, rectal prolapse and rectal bleeding from 7 litters of *Rag1*$^{-/-}$*Smarca4*$^{f/f}$ and *Rag1*$^{-/-}$*Smarca4*$^{\Delta ILC3}$ mice of 6-20-week-old. (B-E) 6-10-week-old *Rag1*$^{-/-}$*Smarca4*$^{f/f}$ and *Rag1*$^{-/-}$*Smarca4*$^{\Delta ILC3}$ mice were sacrifice for analyses. (n=8; Representative of 3 experiments) Frequencies (B) and total numbers (C) of neutrophils in the SI LPLs. (D and E) Frequencies (D) and total numbers (E) of eosinophils in the LI LPLs. (F and G) LI LPLs were isolated from *Smarca4*$^{f/f}$ or *Smarca4*$^{\Delta ILC3}$ mice.（n=6~7，Representative of 3 experiments）(F) Representative flow cytometry plots for neutrophils (CD11b^{+}Ly-6G^{+}) gated on live cells. (G) Frequencies of eosinophils in the large intestinal LPLs. (A-G) Data are represented as means ± SEM. Error bars show SEM.

Supplementary Figure 5 Gene ontology analysis of significantly changed genes in Brg1-deficient ILC3s

Bone marrow chimeric mice were constructed as in Figure S2. ILC3s of the same recipients from the *Rag1*$^{-/-}$*Smarca4*$^{f/f}$(WT) and *Rag1*$^{-/-}$*Smarca4*$^{\Delta ILC3}$(KO) donor origin were sorted from the small intestinal LPLs for RNA-seq analysis. (A) Gene ontology enrichment analyses were performed on downregulated genes and upregulated genes of more than $2^{0.5}$ fold in Brg1-deficient ILC3s, and representative enriched biological processes were shown. (B and C) Representative list of downregulated genes (B) or upregulated genes (C) categorized by gene ontology in Brg1-deficient ILC3s were shown with Z-normalized heatmap.

Supplementary Figure 6 Brg1 suppresses GM-CSF expression in ILC3s in a cell-intrinsic manner

(A-C) SI and LI LPLs isolated from *Smarca4*$^{f/f}$ or *Smarca4*$^{\Delta ILC3}$ mice with PBS, IL-1β (10ng/ml) or TL1A (10ng/ml) overnight or phorbol 12-myristate 13-acetate (PMA) plus ionomycin (P+I) for 3 hours prior to analyses. (A) Representative flow cytometry plots for GM-CSF expression gated on ILC3s (Lin^{-}CD45^{low}Thy1.2^{high}) in SI LPLs. (B and C) Frequencies of GM-CSF^{+} cells in ILC3s from SI (B) and LI (C) LPLs. (n=5~8, Representative of 5 experiments). (D-F) Bone marrow chimeric mice were constructed as in Figure S2. SI and LI LPLs from recipient mice were isolated for analyses 6 weeks later. LPLs were stimulated with PBS, IL-23 (10ng/ml), IL-1β (10ng/ml) or TL1A (10ng/ml) overnight or PMA plus ionomycin for 3 hours prior to analyses (n=6~12, Representative of 4 experiments).

(D) Representative flow cytometry plots showing expression of GM-CSF gated on ILC3s from the small intestine of indicated hosts identified by the congenic marker Thy1.1. (E and F) Frequencies of GM-CSF$^+$ cells in ILC3s from the SI (E) and LI (F) LPLs. (G and H) 4-6-week-old *Rag1*$^{-/-}$*Smarca4*$^{f/f}$ and *Rag1*$^{-/-}$*Smarca4*$^{\Delta ILC3}$ mice were treated with 250ug of IgG or α-GM-CSF per mouse every 3 days for 14 days before analyses. (n=12~14, Representative of 4 experiments) (G) Representative flow cytometry plots for eosinophils (CD11b$^+$Siglec-F$^+$) gated on live cells in LI LPLs. (H) Frequencies and total numbers of eosinophils in the LI LPLs. (A-H) Data are represented as means ± SEM. Error bars show SEM. *P<0.05, **P<0.01, ***P<0.001, ****P<0.0001. Lin$^-$, CD3ε$^-$B220$^-$CD11b$^-$CD11c$^-$.

Supplementary Figure 7 Enhanced IL-17F production by brg1-deficient ILC3s is not a key pathogenic factor in *Rag1*$^{-/-}$*Smarca4*$^{\Delta ILC3}$ mice

(A-C) SI and LI LPLs isolated from *Rag1*$^{-/-}$*Smarca4*$^{f/f}$ and *Rag1*$^{-/-}$*Smarca4*$^{\Delta ILC3}$ mice were stimulated with PBS, IL-23 (10ng/ml), IL-1β (10ng/ml) or TL1A (10ng/ml) overnight or PMA plus ionomycin for 3 hours prior to analyses. (n=5; Representative of 2 experiments) (A) Representative flow cytometry plots for IL-17F expression gated on ILC3s (Lin$^-$ CD45^{low}Thy1.2^{high} RORγt$^+$) in SI LPLs. (B and C) Frequencies of IL-17F$^+$ cells in ILC3s (CD45^{low}Thy1.2^{high}RORγt$^+$) from SI (B) and LI (C) LPLs. (D-G) 4-6-week-old *Rag1*$^{-/-}$*Smarca4*$^{f/f}$ and *Rag1*$^{-/-}$*Smarca4*$^{\Delta ILC3}$ mice were treated with 250ug of IgG or α-IL-17F per mouse every 3 days for 14 days before analyses. (n=6; Representative of 2 experiments) (D) Representative H&E staining of colon sections (magnification 10×). Scale bar is 100μm. (E) Histological scores of colon sections. (F) Representative flow cytometry plots for neutrophils

($CD11b^+Ly6G^+$) gated on live cells in LI LPLs. (G) Frequencies and total numbers of lamina propria infiltrating neutrophils. (A-G) Data are represented as means±SEM. Error bars show SEM. *P<0.05, **P<0.01, ***P<0.001, ****P<0.0001. Lin^-, $CD3\varepsilon^-B220^-CD11b^-CD11c^-$; Data are representative of 2 independent experiments.

Supplementary Figure 8 Brg1 regulates the chromatin accessibility of target genes in ILC3s

ILC3s ($Thy1.2^{high}CD45^{low}$ cells) were purified from the small intestine of *Rag1*$^{-/-}$*Smarca4*$^{f/f}$ or *Rag1*$^{-/-}$*Smarca4*$^{\Delta ILC3}$ mice and ATAC-seq was performed and analyzed. (A) Correlation analysis was performed on Log2fold of gene expression (Brg1-deficient over wild-type ILC3s) and the average Log2fold in accessibility of OCRs distributed at intergenic regions. (B-E) Integrated visualization of ATAC-seq peaks in WT or Brg1-deficient ILC3s, together with published ChIP-seq data of small intestinal ILC3s (GSE85156). Red boxes and green boxes highlight representative WT-ILC3-OCRs and Brg1-KO-ILC3-OCRs respectively. Definition of OCRs was described in methods. R^2 indicate correlation coefficient.

Supplementary Data 1

List of genes used for gene ontology analyses in Figure S5.

Supplementary Data 2

WT-ILC3-OCRs and Brg1-KO-ILC3-OCRs.

Biological triplicates of 50000 ILC3s ($Thy1.2^{high}CD45^{low}$ cells) sorted by flow cytometry from the small intestine of 7-9 week old *Rag1*$^{-/-}$*Smarca4*$^{f/f}$ or *Rag1*$^{-/-}$*Smarca4*$^{\Delta ILC3}$ mice were

subjected to ATAC-seq analysis. Lists of WT-ILC3-OCRs and Brg1-KO-ILC3-OCRs were identified using DEseq2 analysis and shown (see methods). Locations of peaks were shown. RRB_KO1_lg, RRB_KO2_lg and RRB_KO3_lg were the Log_{10}FPKM value of counts from Brg1-KO-ILC3s, and RRB_WT1_lg, RRB_KO2_lg and RRB_KO3_lg were the Log_{10}FPKM value of counts from WT-ILC3s. Log2FoldChange indicate relative ratio of counts from Brg1-KO-ILC3s over WT-ILC3s.

Supplementary Data 3

Enriched motifs in WT or Brg1-KO-ILC3-OCRs.

Motif enrichment analysis on WT-ILC3-OCRs and Brg1-KO-ILC3-OCRs were analyzed using Homer2. Representative enriched motifs were listed with cut off p value of 1×10^{-6}.

Supplementary Table 1. List of antibodies used for flow cytometry

Clone numbers and manufactures of the antibodies used for flow cytometry were shown.

Antibody	Clone	Company
CD3e	145-2C11	Thermo Fisher Scientific
B220	RA3-6B2	Thermo Fisher Scientific
CD11b	M1/70	Thermo Fisher Scientific
CD11c	N418	Thermo Fisher Scientific
CD4	RM4-5	Thermo Fisher Scientific
Foxp3	FJK-16s	Thermo Fisher Scientific
CD62L	MEL-14	Thermo Fisher Scientific
CD44	IM7	Thermo Fisher Scientific
Ly-6G	1A8	BD Pharmingen
Siglec-F	E50-2440	BD Pharmingen
NKp46	29A1.4	Thermo Fisher Scientific
IL-17F	O79-289	BD Pharmingen
GM-CSF	MP1-22E9	ebioscience
CD103	2E7	Thermo Fisher Scientific
RORγt	B2D	Thermo Fisher Scientific
Thy-1.2	30-H12	Thermo Fisher Scientific
Thy-1.1	HIS51	Thermo Fisher Scientific
CD45	30-F11	Thermo Fisher Scientific
CCR6	140706	BD biosciences
T-bet	eBio4B10 (4B10)	Thermo Fisher Scientific
Ki-67	SolA15	Thermo Fisher Scientific
CD16/CD32	93	Thermo Fisher Scientific

Supplementary Table 2. List of primers used for quantatative PCR

Sequences for primers used for real-time RT-PCR or ChIP-Q-PCR experiments were shown.

Gene or Locus	Forward Primer	Reverse Primer
Smarca4	CAAAGACAAGCATATCCTAGCCA	CACGTAGTGTGTGTTAAGGACC
Dll1	GGGGACAGAGGGGAGAAGAT	AGCCTTCCTGGCAGTTACAC
Hes1	CAACACGACACCGGACAAAC	GGAATGCCGGGAGCTATCTT
Rbpj	TCCATCGGCGGGGAAGTT	ACTGTTTGATCCCCTCGTTCTT
Notch2	CAGCACTGTGACAGCCCTTA	TATTCCGCTCACAGGTGCTC
Tbx21	AAGTTCAACCAGCACCAGACA	GGTGGACATATAAGCGGTTCC
Csf2	TCAAGAAGCTAACATGTGTGCAG	ACAGTCCGTTTCCGGAGTTG
Il17f	CCCAGGAAGACATACTTAGAAGAAA	CAACAGTAGCAAAGACTTGACCA
Tbx21 promoter	CACACAGACACCGGTTGGTA	TGGGGTAGGCTGGGGAAATA
Tbx21.1	ATAGGCAGGTGGGTCTGACA	AACAAGGGGGCTTCCAACAA
Csf2 promoter	TGTCATTCTCACTGCTCCCAAG	GGAGACCATTCTGGGCAAACT
Csf2.1	CCTAACCTGGCAGAAGAGCC	TGTCCAGAGAGATGCTCCCA
Csf2 CNSc	GGCTTCAGCTCCCCACTTCT	GGTTTTCTAGCTTATATCCCCAGACA
